# Habitual Daily Intake of Fried Foods Raises Transgenerational Inheritance Risk of Heart Failure Through NOTCH1-Triggered Apoptosis

**DOI:** 10.34133/research.0401

**Published:** 2024-07-15

**Authors:** Anli Wang, Xuzhi Wan, Fanghuan Zhu, Haoyin Liu, Xiaoran Song, Yingyu Huang, Li Zhu, Yang Ao, Jia Zeng, Binjie Wang, Yuanzhao Wu, Zhongshi Xu, Jiye Wang, Weixuan Yao, Haoyu Li, Pan Zhuang, Jingjing Jiao, Yu Zhang

**Affiliations:** ^1^ Department of Gastroenterology, The First Affiliated Hospital, Zhejiang University School of Medicine; Zhejiang Key Laboratory for Agro-Food Processing, College of Biosystems Engineering and Food Science, Zhejiang University, Hangzhou, Zhejiang, China.; ^2^ Department of Endocrinology, The Second Affiliated Hospital, Department of Nutrition, School of Public Health, Zhejiang University School of Medicine, Hangzhou, Zhejiang, China.; ^3^ Key Laboratory of Drug Prevention and Control Technology of Zhejiang Province, Department of Criminal Science and Technology, Zhejiang Police College, Hangzhou, Zhejiang, China.

## Abstract

Consumption of fried foods is highly prevalent in the Western dietary pattern. Western diet has been unfavorably linked with high risk of developing cardiovascular diseases. Heart failure (HF) as a cardiovascular disease subtype is a growing global pandemic with high morbidity and mortality. However, the causal relationship between long-term fried food consumption and incident HF remains unclear. Our population-based study revealed that frequent fried food consumption is strongly associated with 15% higher risk of HF. The causal relationship may be ascribed to the dietary acrylamide exposure in fried foods. Further cross-sectional study evidenced that acrylamide exposure is associated with an increased risk of HF. Furthermore, we discover and demonstrate that chronic acrylamide exposure may induce HF in zebrafish and mice. Mechanistically, we reveal that acrylamide induces energy metabolism disturbance in heart due to the mitochondria dysfunction and metabolic remodeling. Moreover, acrylamide exposure induces myocardial apoptosis via inhibiting NOTCH1-phosphatidylinositol 3-kinase/AKT signaling. In addition, acrylamide exposure could affect heart development during early life stage, and the adverse effect of acrylamide exposure is a threat for next generation via epigenetic change evoked by DNA methyltransferase 1 (DNMT1). In this study, we reveal the adverse effects and underlying mechanism of fried foods and acrylamide as a typical food processing contaminant on HF from population-based observations to experimental validation. Collectively, these results both epidemiologically and mechanistically provide strong evidence to unravel the mechanism of acrylamide-triggered HF and highlight the significance of reducing fried food consumption for lower the risk of HF.

## Introduction

Heart failure (HF), an important cardiovascular disease (CVD) subtype, is a growing clinical pandemic and drives heavy healthcare expenditures globally [1], affecting an estimated 64.3 million people worldwide [2]. While any condition disrupting myocardial function can lead to HF [3], the habitual consumption of unhealthy diet including Western diet has recently been linked with high risk of developing HF [4,5]. Fried foods, a popular choice globally, saw heightened consumption during the COVID-19 pandemic [6]. So far, only limited evidence has supported fried food consumption in relation to higher risk of HF among US male physicians [7], which propelled further concerns about causal relationship among general population. Despite fascinating flavor and unique texture, the frying process has the potential to generate hazardous chemicals through thermal reactions involving various ingredients and nutrients. Acrylamide, identified as a representative hazardous contaminant in fried foods, is produced primarily through the Maillard reaction, particularly in carbohydrate-rich foods [8]. Daily exposure to acrylamide from fried foods, from infants to the elderly, remains an important public health concern [9]. In particular, dietary intake of acrylamide has been associated with an increased risk of mortality [10], which drives the motivation to address diet-sourced acrylamide as a potential contributor for linking fried food consumption with incident HF, a leading cause of death in CVD events. However, unfortunately, the causation of acrylamide-triggered HF has not been well understood.

To unravel the etiology, mitochondrial dysfunction has been implicated in HF development [11]. Mitochondrial oxidative metabolism and continuous adenosine 5′-triphosphate (ATP) production are essential for maintaining normal cardiac function [11–13]. Fatty acid oxidation diminishes in the failing heart due to transcriptional changes in fatty acid oxidation enzymes and transporters [12,14]. Apoptosis contributes to cardiomyocyte loss in HF, involving cytochrome C release and caspase 3 activation [15]. Although several cellular-level studies revealed mitochondrial toxicity of acrylamide [16,17], the mechanistic role of acrylamide in mitochondrial dysfunction and cardiomyocyte apoptosis still remains obscure. In addition, the NOTCH signaling pathway, vital in cardiac development, reduces cardiomyocyte apoptosis [18–21]. NOTCH signaling, activated by the phosphatidylinositol 3-kinase (PI3K)/AKT pathway after myocardial injury, supports a positive survival feedback with PI3K/AKT signaling [22]. Notably, our previous study indicated that acrylamide exposure disrupted Notch signaling dynamics during early cardiogenesis in zebrafish embryos, raising the intriguing possibility that acrylamide may induce HF via mitochondrial dysfunction and NOTCH-triggered cardiomyocyte apoptosis.

DNA methylation is a critical epigenetic modification that regulates numerous biological processes essential for cardiac function, and dysregulation has consistently been linked with HF [23,24]. Previous study suggest the impact of acrylamide exposure on oocyte quality and sperm parameters, involving reactive oxygen species (ROS) generation, apoptosis induction, and epigenetic modifications [25]. In addition, NOTCH pathway is activated by irreversible CpG DNA methylation in adult cardiomyocytes after myocardial infarction [26]. However, it is very little known about the role of DNA methylation in regulating the development of acrylamide-induced HF during early life stages.

Given high fried food consumption worldwide, here, we sought to understand how dietary-sourced acrylamide induces HF and further transgenerational inheritance through mitochondrial dysfunction and NOTCH-signaling-triggered apoptosis.

## Results

### Fried food consumption is associated with higher risk of HF

**Fig. 1.**
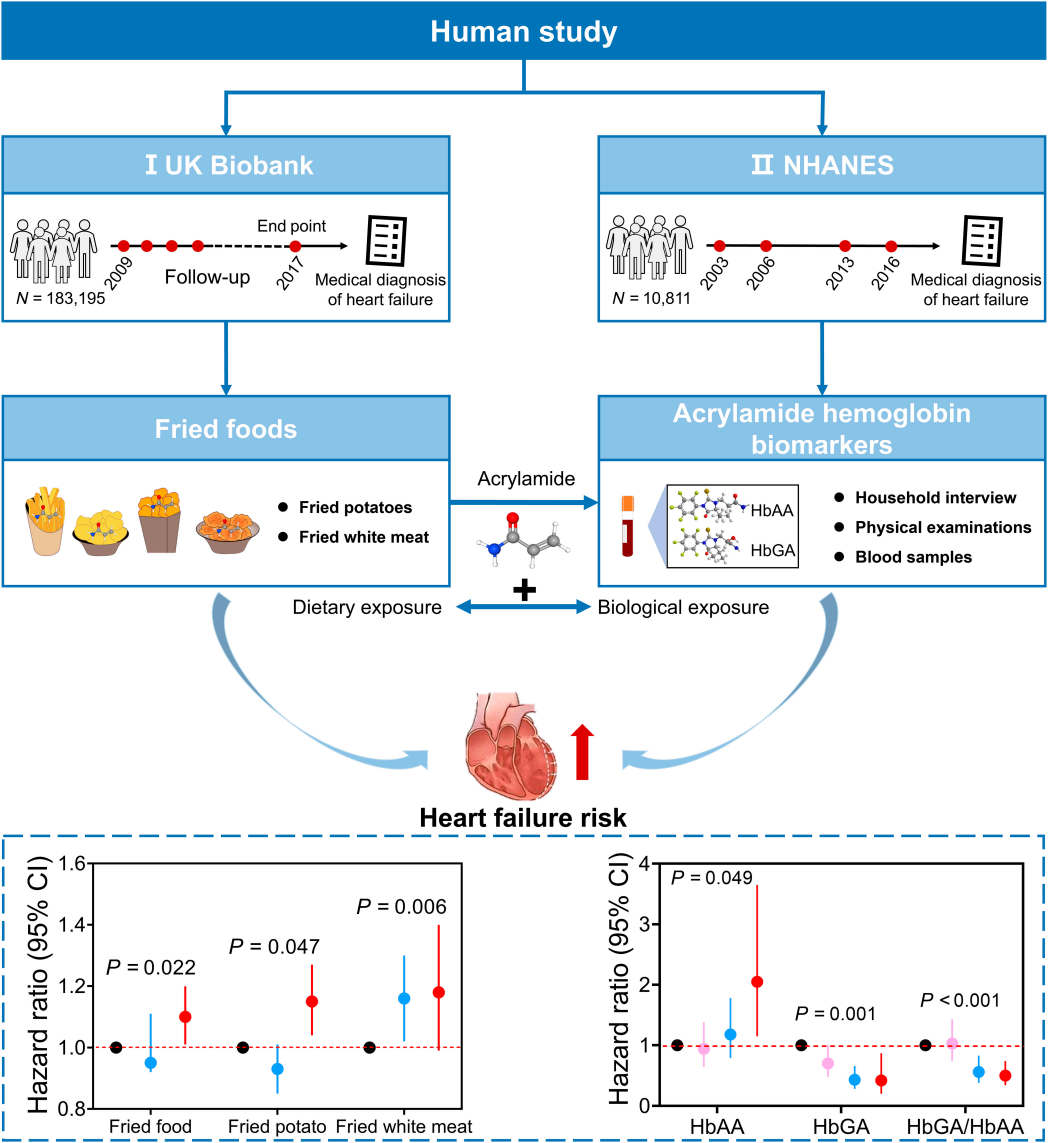
Study design and outcomes for the associations of fried food consumption or hemoglobin biomarkers of acrylamide with the risk of HF in the UK Biobank and NHANES.

To assess the association of fried food consumption with HF, we included 183,195 participants from the UK Biobank study, excluding those with invalid diet records, HF, cancer, and/or other cardiovascular health conditions at baseline (Fig. 1 and Fig. S1). A total of 3,012 HF cases were identified during an average of 12.2 years of follow-up. We then profiled baseline characteristics of enrolled participants according to fried food consumption (Table S1), revealing that individuals consuming over one serving per day were more likely to be male, younger, and active smokers, with higher body mass index, lower household income, less use of vitamin and mineral supplements, and higher energy intake. Similar trends were observed for fried potato and fried white meat consumption (Tables S2 and S3). Cox proportional hazard regression analyses, adjusted for multiple variables, demonstrated a 10% higher HF risk associated with fried food consumption [hazard ratio, 1.10; 95% confidential interval (CI), 1.01 to 1.20; *P* = 0.022 for trend]. Consistently, fried white meat and fried potato consumption were significantly linked to an 18% and 15% higher HF risk, respectively (Table S4). Subgroup analyses indicated stronger associations among male, participants older than 60 years, and nonsmokers (Fig. S2). Sensitivity analyses confirmed the robustness of the positive association for fried food consumption even after further controlling for the history of type 2 diabetes, CVD, vitamin, and mineral supplementation or medication use or further excluding the participants with incident HF within 5 years or missing covariate data at baseline (Table S5).

### Acrylamide in fried foods mainly contributes to the association with higher risk of HF

Commercial fried foods contain various hazardous chemical contaminants produced during thermal processing. Acrylamide as a typical contaminant conjugates with hemoglobin for a long-term exposure to induce potential health risk [8,27]. To further assess whether acrylamide mediates the association, we next investigated whether long-term exposure to acrylamide in the forms of hemoglobin adducts is associated with the prevalence of HF among general people from the National Health and Nutrition Examination Survey (NHANES). We finally enrolled 10,811 eligible people after excluding participants who were younger than 20 years, without data on hemoglobin adduct levels of acrylamide [hemoglobin adduct of acrylamide (HbAA) and hemoglobin adduct of glycidamide (HbGA)] and with incomplete dietary data (Fig. 1 and Fig. S3), and summarized baseline characteristics categorized by HbAA and HbGA levels (Table S6). To address concerns about hemoglobin exposure, multivariate-adjusted logistic regression analyses were conducted, confirming the mediating role of acrylamide. HbAA was associated with a higher HF prevalence [odds ratio (OR) (95% CI), 2.05 (1.15 to 3.65)] (Fig. 1 and Table S7). Consistently, the HbGA/HbAA ratio showed an inverse association with HF prevalence [OR (95% CI), 0.50 (0.34 to 0.74)]. This provides robust evidence supporting the positive association between hemoglobin adducts of acrylamide and HF prevalence, suggesting that acrylamide mediate the previously observed link between frequent fried food consumption and an increased risk of HF.

### Long-term exposure to acrylamide induces HF

**Fig. 2.**
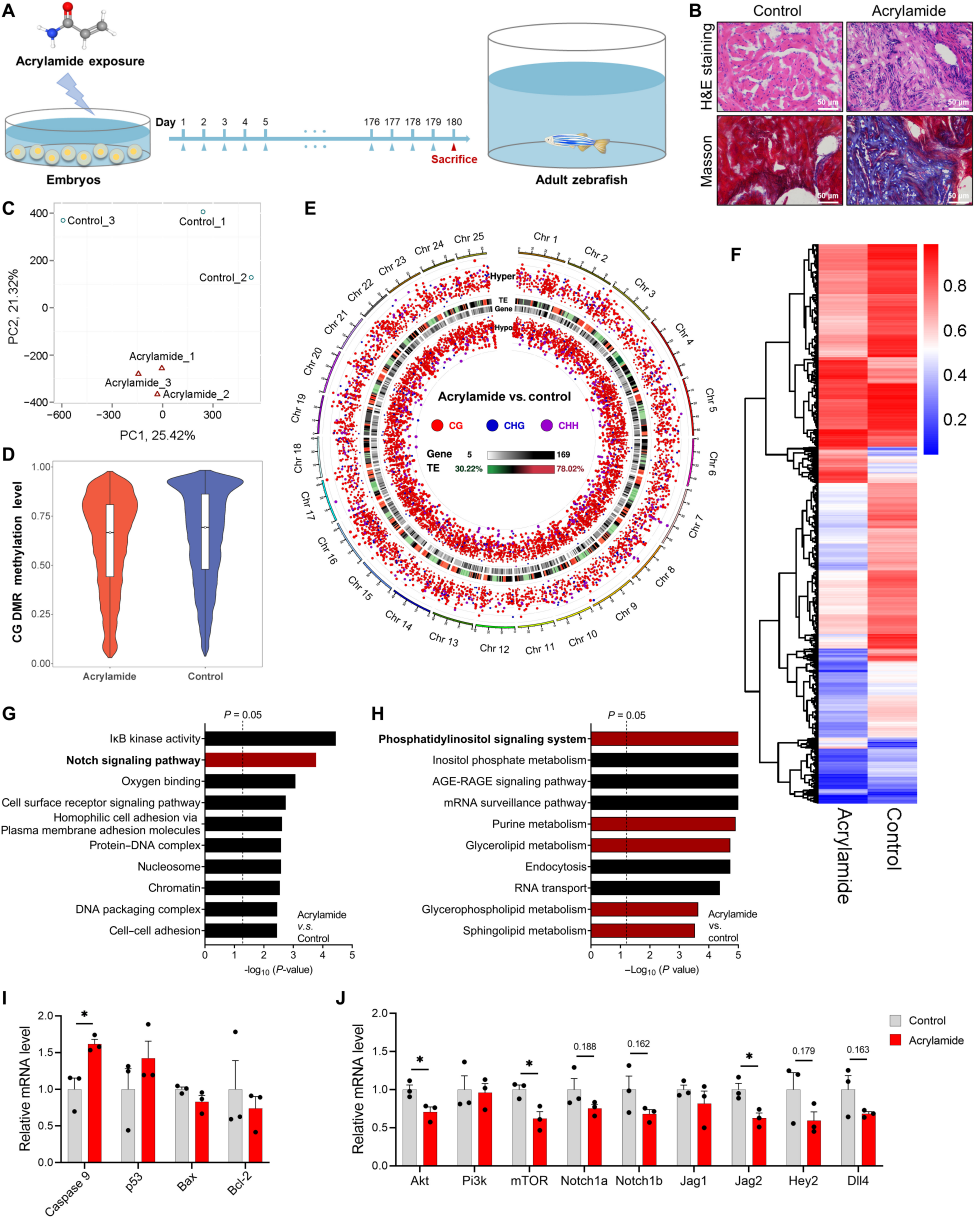
Chronic exposure to acrylamide induces HF in adult zebrafish. (A) Experimental design: embryos at 2 hpf exposed to 0.25 mM acrylamide for 180 d. (B) Representative images of hematoxylin and eosin (H&E) and Masson staining of adult zebrafish heart sections in control and acrylamide treatment (0.25 mM) groups. (C) The control and acrylamide-treated groups were completely distinguished by different colors in principal components analysis (PCA) plots based on WGBS analysis. (D) The global methylation level between the control and acrylamide-treated groups based on WGBS analysis. (E) Global methylation patterns in zebrafish hearts exposed to 0.25 mM acrylamide. (F) Heatmap showing differentially methylated changes in heart tissue between control and acrylamide-treated zebrafish based on CG pattern. (G and H) GO and KEGG analyses of WGBS data. (I) Relative mRNA expression of cardiac-apoptosis-related genes in control and acrylamide-treated zebrafish (*n* = 3 per group). (J) Relative mRNA expression of cardiac NOTCH and PI3K/AKT-related genes in control and acrylamide-treated zebrafish (*n* = 3 per group). Data are presented as the means ± SEM. Significance was calculated using 2-tailed *P* values by unpaired Student’s *t* test; *P < 0.05.

**Fig. 3.**
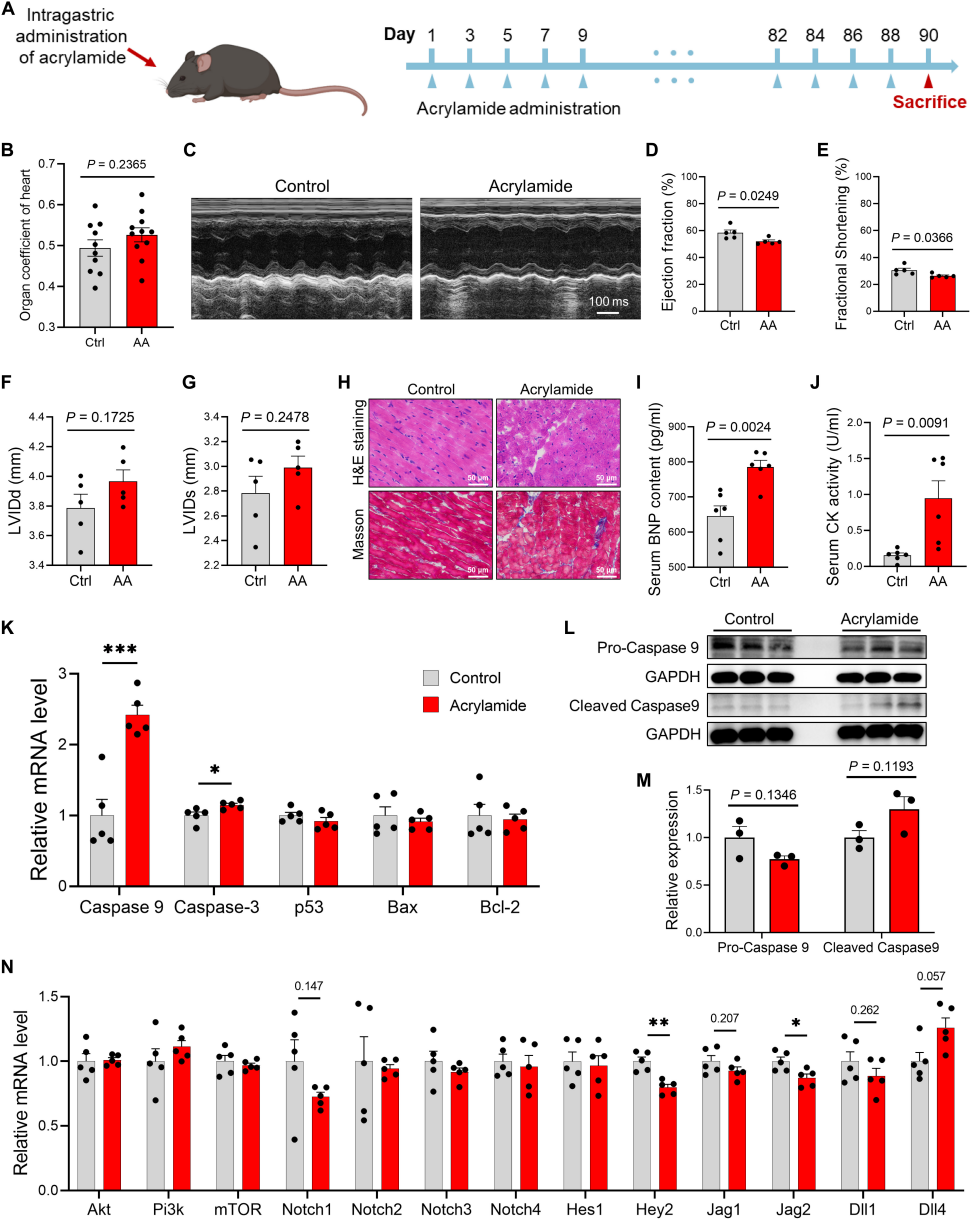
Chronic exposure to acrylamide exposure induces HF in mice. (A) Experimental design: 8-week-old mice exposed to acrylamide (0.5 mg/kg·bw per day) for 90 d. (B) The organ coefficient of the heart in control and acrylamide treatment groups. (C) Representative M-mode echocardiogram in control and acrylamide treatment groups. (D) Ejection fraction (%), (E) fractional shortening (%), (F) left ventricular internal dimension diastole (LVIDd) (in millimeters), and (G) left ventricular internal dimension systole (LVIDs) (in millimeters) based on echocardiogram results in control and acrylamide (AA) treatment groups (*n* = 5 per group). (H) Representative images of H&E and Masson staining of mouse heart sections in control and acrylamide treatment groups (0.5 mg/kg·bw per day). (I) Serum BNP level and (J) serum CK level in control and acrylamide treatment groups (*n* = 6 per group). (K to M) Relative mRNA expression of cardiac apoptosis-related genes and proteins in control and acrylamide-treated mice (*n* = 5 per group for quantitative polymerase chain reaction and *n* = 3 per group for Western blotting). (N) Relative mRNA expression of cardiac NOTCH and PI3K/AKT-related genes in control and acrylamide-treated mice (*n* = 5 per group). Data are presented as the means ± SEM. Significance was calculated using 2-tailed *P* values by unpaired Student’s *t* test; *P < 0.05, **P < 0.01, and ***P < 0.001.

To unravel the causation, we delved into how chronic exposure to acrylamide triggers HF (Figs. 2 and 3). Zebrafish subjected to aquatic exposure to acrylamide (0.25 mM) for 180 d morphologically caused infiltration of inflammatory cells, myocyte damage, and excessive collagen deposition (Fig. 2A and B), indicating significant cardiomyocyte damage and cardiac fibrosis, a classic pathologic HF feature. To gain further insight into epigenetic changes, we performed whole-genome bisulfite sequencing (WGBS) analysis for adult zebrafish heart and observed lower global methylation level in acrylamide-exposed group compared with the control group (Fig. 2C and D). Total differential methylation and both hypermethylation and hypomethylation seemed variable between chromosomes (Fig. S4A). In detail, we found that long-term exposure to acrylamide declines DNA methylation levels of exon, utr5, CpG island shore, and CpG island (Fig. S4B to E). We further conducted differentially methylated region (DMR) identification and regional annotations and recognized 17,680 DMRs including 7,133 hypermethylated and 10,547 hypomethylated in hearts of adult zebrafish between the 2 groups (Fig. 2E and Fig. S4F to H), which was confirmed by the DMR expression heatmap (Fig. 2F). The Gene Ontology (GO) enrichment analysis revealed these DMRs function in inhibitor of nuclear factor κB (IκB) kinase activity, NOTCH signaling pathway, oxygen binding, etc. (Fig. 2G). Meanwhile, the Kyoto Encyclopedia of Genes and Genomes (KEGG) pathway analysis highlighted enrichment in the phosphatidylinositol signaling system, purine metabolism, glycerolipid metabolism, glycerophospholipid metabolism, and sphingolipid metabolism (Fig. 2H). Interestingly, we found that the mRNA expression of *Caspase 9*, but not *P53*, *Bax*, and *Bcl-2*, increased in the acrylamide-exposed group (Fig. 2I). Furthermore, long-term exposure to acrylamide led to a decrease in the expression of genes involved in the regulation of the PI3K/AKT and NOTCH signaling pathways (Fig. 2J).

To gain insights into HF, we had served mice (C57BL/6) with a 90-d chronic exposure to acrylamide [0.5 mg/kg·body weight (bw) per day] (Fig. 3A). Subsequent validation indicated a slight increase in the organ coefficient of the heart compared to the control group (Fig. 3B). Echocardiography, used to measure heart function, unveiled diminished systolic function and an increased chamber diameter in the hearts of acrylamide-exposed mice (Fig. 3C to G and Movie S1). Consistently, histologic observations indicated chronic exposure to acrylamide caused infiltration of inflammatory cells, myocyte damage, and increased myocardial fibrosis (Fig. 3H), indicating significant cardiomyocyte damage and cardiac fibrosis. To further validate by diagnosed biomarkers of HF, we found that serum brain natriuretic peptide (BNP) and creatine kinase (CK) levels were significantly elevated because of acrylamide exposure (Fig. 3I and J). In line with this, the mRNA expression of both *Anp* and *Bnp*, 2 HF markers, was also sharply enhanced (Fig. S4I and J). Notably, we found increased expression of *Tnf-α*, *IL1-β*, and *IL-6* by acrylamide exposure (Fig. S4K), indicating the cardiac inflammatory response. To confirm whether acrylamide-induced HF is driven by cardiac apoptosis, we showed that acrylamide significantly elevates the expression of *Caspase 9* and *Caspase 3*, but not *P53*, *Bax*, and *Bcl-2*, in hearts (Fig. 3K), which was supported by previous zebrafish results. Further study consistently evidenced down-regulated protein expression of pro-Caspase 9 but up-regulated cleaved Caspase 9 (Fig. 3L and M). In addition, long-term exposure to acrylamide disturbs NOTCH pathway via *Notch1*, *Jag1*, *Jag2*, *Dll1*, and *Dll4* and the downstream signal *Hey2* (Fig. 3N). Together, these results support the evidence of acrylamide-induced HF and suggest that dysregulation of NOTCH signaling and NOTCH-triggered cardiac apoptosis may be a relevant mechanism.

### Chronic exposure to acrylamide disturbs cardiac energy metabolism due to mitochondrial dysfunction and metabolic remodeling

**Fig. 4.**
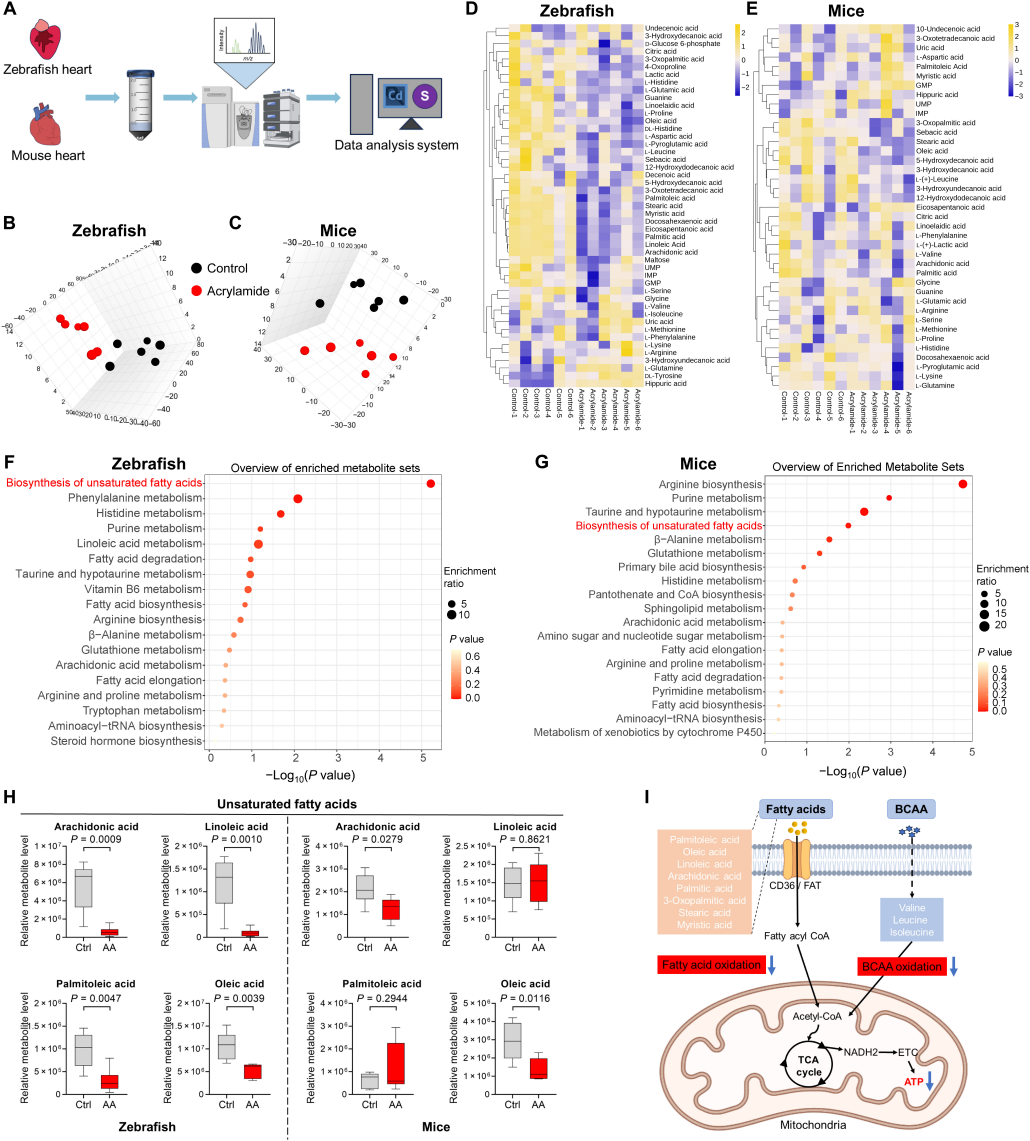
Chronic exposure to acrylamide induces mitochondria dysfunction and metabolic remodeling based on metabolomics analysis in zebrafish and mice. (A) Schematic diagram of metabolomics for the isolated hearts from both zebrafish and mice. (B and C) 3-dimensional PCA scores plots of metabolites profiles of hearts from zebrafish and mice exposed to acrylamide, respectively (*n* = 6 per group). (D and E) Heatmap of metabolites based on the normalized mass spectrometry intensity in hearts of zebrafish and mice, respectively. (F and G) KEGG pathway analysis of differential metabolites in hearts of zebrafish and mice, respectively. (H) The relative unsaturated fatty acids levels in hearts of zebrafish and mice, respectively. (I) Model of cardiac mitochondrial energy metabolism disturbance induced by chronic acrylamide exposure. The illustration was created using BioRender. Data are presented as the means ± SEM. Significance was calculated using 2-tailed *P* values by unpaired Student’s *t* test. *m*/*z*, mass/charge ratio; CoA, coenzyme A; UMP, uridine monophosphate; GMP, guanosine monophosphate; IMP, inosine monophosphate; BCAA, branched-chain amino acid; NADH2, nicotinamide adenine dinucleotide 2; ETC, electron transfer chain; TCA, tricarboxylic acid.

HF involves cardiac mitochondrial dysfunction and metabolic remodeling [12,28], but the toxic role of acrylamide in this underlying mechanism remains unknown. As expected, energy metabolism of mitochondria-related GO terms was finally filtered on the basis of RNA sequencing (RNA-seq) data from hearts of rat embryos, indicating that acrylamide may drive the changes in cardiac energy metabolism (Fig. S4L). To further confirm this, we performed untargeted metabolomics of isolated hearts from both adult zebrafish and mice to identify metabolites involved in mitochondrial dysfunction and disturbed pathways (Fig. 4A). The resulting metabolic profiles were clearly segregated between the control and acrylamide treatment groups (Fig. 4B and C and Fig. S5). In total, 47 and 39 differential metabolites were identified from 3,824 and 1,980 metabolites in hearts of adult zebrafish and mice, respectively (Fig. 4D and E). Notably, we used the KEGG library to enrich the top 25 metabolic pathways, including biosynthesis of unsaturated fatty acids, histidine metabolism, purine metabolism, taurine and hypotaurine metabolism, and arginine biosynthesis as the common enrichment pathways in hearts of both animals (Fig. 4F and G and Fig. S6). In line with this, we then established global metabolic pathway networks (Fig. S7) and identified a range of metabolites that were involved and significantly reduced in acrylamide-induced HF, including saturated fatty acids (palmitic acid, 3-oxopalmitic acid, stearic acid, and myristic acid), unsaturated fatty acids [arachidonic acid, linoleic acid, docosahexaenoic acid, eicosapentanoic acid, palmitoleic acid, oleic acid, (2E)-decenoic acid, and 10-undecenoic acid], and fatty acid ω-oxidation products (sebacic acid, 12-hydroxydodecanoic acid, 3-oxotetradecanoic acid, and 5-hydroxydecanoic acid) (Fig. 4H and Fig. S8). Considering that energy metabolic changes in HF often involve reductions in mitochondrial fatty acid oxidation (Fig. 4I and Fig. S8) [29], indicating the deficiency of adequate energy supply to the heart, these results demonstrated acrylamide disturbs cardiac energy metabolism due to mitochondrial dysfunction and metabolic remodeling (Fig. S9A).

### Acrylamide induces mitochondrial damage and myocardial apoptosis

**Fig. 5.**
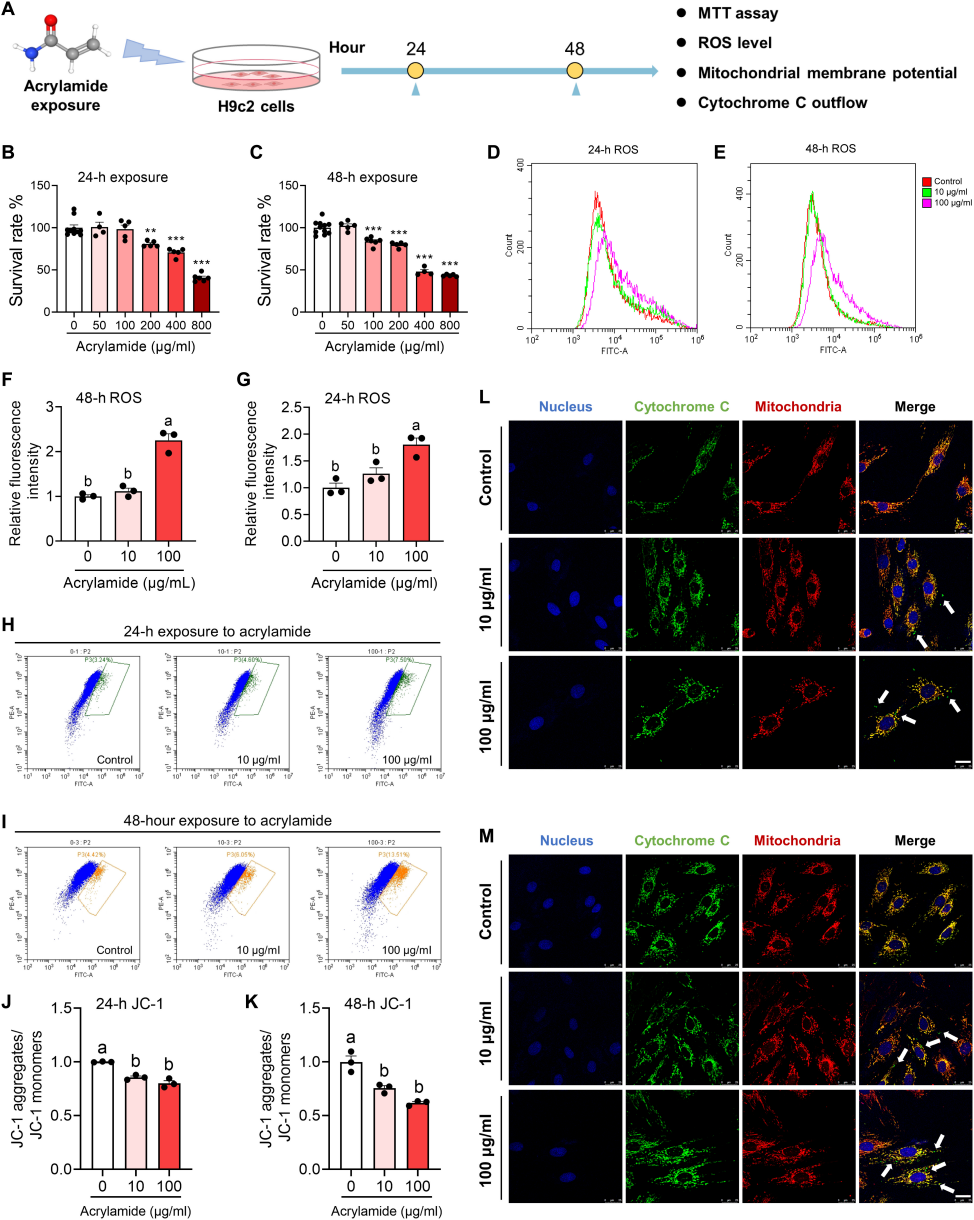
Acrylamide induces cardiomyocyte apoptosis with ROS accumulation, cytochrome C outflow, and dropped mitochondrial membrane potential in H9c2 cells. (A) Schematic diagram of cell study. (B and C) Survival rates of H9c2 cells after 24- and 48-h acrylamide exposure, respectively. The viability of cells was determined by the 3-(4,5)-dimethylthiahiazo (-z-y1)-3,5-di-phenytetrazoliumromide (MTT) assay. (D and E) Accumulation of ROS was observed at 24 and 48 h by 2,7-dichlorodihydrofluorescein diacetate (DCFH-DA) probe after acrylamide treatment. (F and G) Quantification of ROS accumulation in H9c2 cells after 24- and 48-h acrylamide exposure, respectively (*n* = 3 per group). (H and I) Mitochondrial membrane potential was evaluated at 24 and 48 h by JC-1 probe after acrylamide treatment. (J and K) Quantification of mitochondrial membrane potential in H9c2 cells after 24- and 48-h acrylamide exposure, respectively (*n* = 3 per group). (L and M) Cytochrome C outflow was observed by colocalizing mitochondria (red) and cytochrome C (green) fluorescently, while cell nucleus was dyed by 4′,6-diamidino-2-phenylindole (DAPI) (blue) with acrylamide exposure for 24 and 48 h, respectively. The white arrows point out the occurrence of cytochrome C outflow. Data are presented as the means ± SEM. Significance was calculated using one-way analysis of variance (ANOVA) with Tukey’s post hoc test; **P < 0.01 and ***P < 0.001; groups labeled with different letters differed significantly (*P* < 0.05). FITC, fluorescein isothiocyanate.

**Fig. 6.**
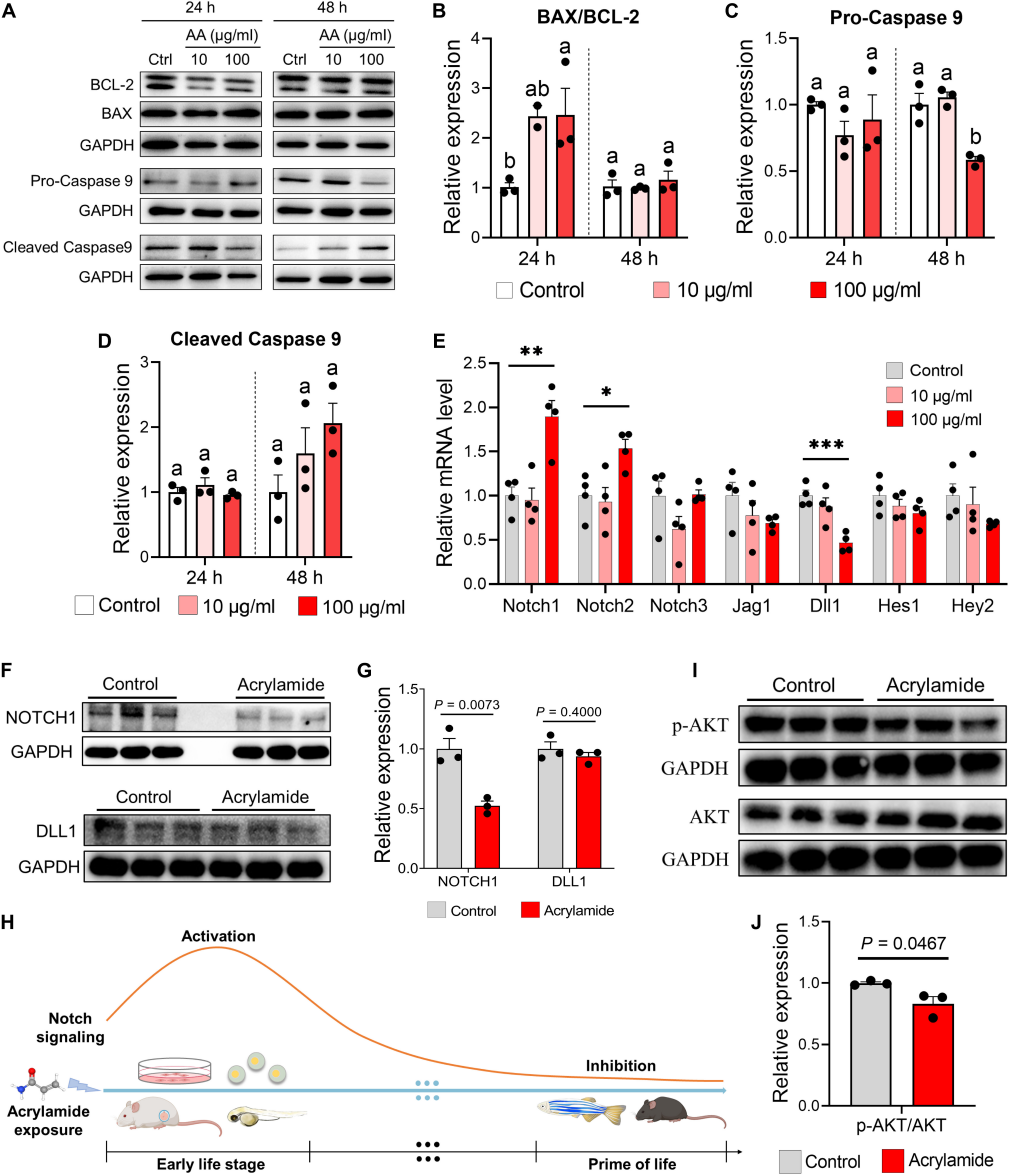
Acrylamide induces cardiomyocyte apoptosis via NOTCH-PI3K/AKT signaling in H9c2 cells. (A to D) Western blotting of apoptotic pathway related proteins (BCL-2, BAX, pro-Caspase 9, and cleaved Caspase 9) shows that acrylamide treatment activates apoptotic pathway in H9c2 cells (*n* = 3 per group). (E) Relative mRNA expression of NOTCH signaling pathway-related genes in H9c2 cells with acrylamide treatment for 48 h (*n* = 4 per group). (F and G) Western blotting of NOTCH1 and DLL1 proteins shows that acrylamide treatment inhibits NOTCH signaling pathway in heart of 5-month-old mice (*n* = 3 per group). (H) Schematic diagram of NOTCH signaling changes with acrylamide exposure. (I and J) Western blotting of p-AKT and AKT proteins shows that acrylamide treatment inhibits PI3K/AKT signaling pathway in heart of mice (*n* = 3 per group). Data are presented as the means ± SEM. Significance was calculated using 2-tailed *P* values by unpaired Student’s *t* test or one-way ANOVA with Tukey’s post hoc test; *P < 0.05, **P < 0.01, and ***P < 0.001; groups labeled with different letters differed significantly (*P* < 0.05). GAPDH, glyceraldehyde-3-phosphate dehydrogenase.

The evidence of acrylamide-induced HF via energy metabolism disturbance raises the possibility that acrylamide may initially trigger mitochondrial dysfunction and activate cellular apoptosis. To explore this hypothesis, we utilized rat embryonic cardiomyocytes (H9c2 cells) to reveal how acrylamide impairs embryonic cardiac development (Fig. 5A) in dose-dependent (10 and 100 μg/ml) and time-dependent (24 and 48 h) ways given the survival rates (Fig. 5B and C and Fig. S10A). The cellular toxicity of acrylamide stems from oxidative stress caused by an imbalance of the biological oxidant to antioxidant ratio [30]. Excessive oxidation may cause the destruction of macromolecules and ultimately lead to cell apoptosis [31]. Consistently, we observed significant accumulation of ROS at 24 h (*P* < 0.01) and 48 h (*P* < 0.001) after high-dose (100 μg/ml) treatment with acrylamide, while in the low-dose (10 μg/ml) group, an upward trend existed although cellular ROS accumulation was not elevated significantly (Fig. 5D to G). Subsequently, the mitochondrial membrane potential dropped significantly at 24 and 48 h in both treatment groups (Fig. 5H to K), indicating disturbed mitochondrial homeostasis. The decrease in mitochondrial membrane potential exacerbates membrane permeability and thus induces cytochrome C outflow [32]. To verify this, we visualized acrylamide-induced cytochrome C outflow by fluorescently colocalizing mitochondria and cytochrome C. Under normal conditions, the signals of mitochondria and cytochrome C overlapped completely (Fig. 5L and M). After 24-h exposure, cytochrome C was released outside the mitochondria (Fig. 5L). Then, the phenomena of cytochrome C outflow seemed more severe after 48-h exposure (Fig. 5M). Apoptotic protease activating factor 1 and outflowed cytochrome C combine to create ATP-mediated apoptotic bodies, which further trigger the downstream apoptotic pathway [33]. Here, 24-h exposure to high-dose acrylamide significantly promoted the expression of apoptosis factor Bcl-2 assaciated X (BAX)/B cell lymphoma 2 (BCL-2) (*P* < 0.05) (Fig. 6A and B), but the expression of both pro-Caspase 9 and cleaved Caspase 9 did not change significantly (Fig. 6C and D), suggesting activation of a mitochondrial-mediated apoptotic pathway after 24-h acrylamide treatment [34]. After 48-h exposure, the expression of BAX/BCL-2 resumed to normal level due to a stress repair effect (Fig. 6B). Nonetheless, high-dose exposure to acrylamide drove significant down-regulation of pro-Caspase 9 and marginally significant up-regulation of cleaved Caspase 9 (Fig. 6C and D), suggesting the activation of Caspase 9-mediated apoptotic pathway. These results demonstrate that acrylamide exacerbates ROS-related mitochondrial dysfunction and energy metabolism disturbance and thus eventually induces mitochondrial damage and activates the apoptosis pathway (Fig. S9B).

### Acrylamide induces cardiomyocyte apoptosis via inhibiting NOTCH1-PI3K/AKT signaling

Cardiomyocyte apoptosis depends on the precise coordination of various signaling pathways, among which, NOTCH signal plays an important role [22,35]. We previously found that acute exposure to acrylamide abnormally activates NOTCH signals in zebrafish heart and exacerbates trabecular muscle hyperplasia and failure of cardiac development from 3 to 14 d postfertilization (dpf) [36]. In our current study, we found that acrylamide treatment (100 μg/ml) significantly promotes cellular mRNA expression of *Notch1* at 24 and 48 h but suppresses the expression of *Dll1* (Fig. 6E and Fig. S10B). Interestingly, long-term and low-dose exposure to acrylamide significantly inhibits the protein expression of NOTCH1 in hearts of 5-month-old mice, while marginally down-regulating the expression of delta-like canonical Notch ligand 1 (DLL1), one of NOTCH1 ligands (Fig. 6F and G). These results indicates that the activation of NOTCH signaling pathway is involved in repair of acute acrylamide-induced myocardial injury at early stages, while chronic exposure to acrylamide induces myocardial apoptosis by inhibiting NOTCH pathway (Fig. 6H) [37]. We previously found that acrylamide interferes with the expression of receptors and ligands in NOTCH signaling and repressed the expression of downstream gene (*Hey2*) (Figs. 2J and 3N). Thus, we further evaluated the expression of phosphorylation of AKT protein and found that reduced expression of S473 phosphorylated AKT with a 48-h acrylamide treatment (100 μg/ml) (Fig. S10C and D). Furthermore, chronic acrylamide exposure inhibits the phosphorylated expression of AKT in hearts of mice (Fig. 6I and J), indicating that acrylamide disturbs noncanonical NOTCH pathway mediated by AKT signals. Taken together, chronic exposure to acrylamide may induce cardiomyocyte apoptosis via inhibiting NOTCH1-PI3K/AKT signaling and mitochondrial dysfunction (Fig. S9B).

### Acrylamide triggers abnormal cardiac development during early life stages

**Fig. 7.**
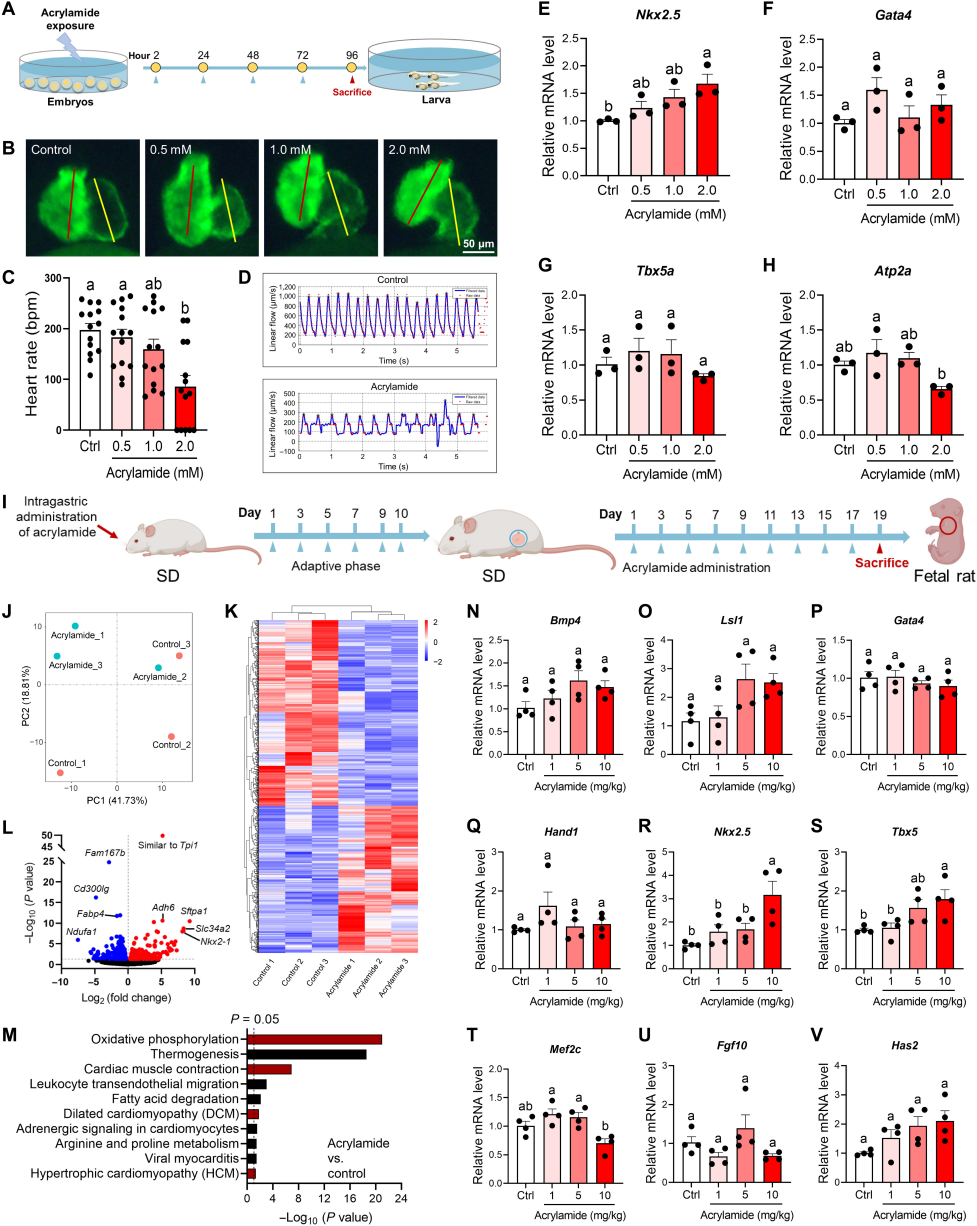
Acrylamide disturbs heart development during early life stage in both zebrafish embryos and rat embryos. (A) Experimental design: embryos exposed to acrylamide (0.5, 1.0, and 2.0 mM) from 2 to 96 hpf. (B) Representative fluorescence microscopy images of *Tg(cmlc2: GFP)* zebrafish embryos with green fluorescent protein specifically expressed in the myocardial cells. Embryos at 2 hpf were exposed to acrylamide (0.5, 1.0, and 2.0 mM) for 4 d. (C) Heart rates were measured in zebrafish (2 hpf) treated with acrylamide for 4 d (*n* = 20 per group). (D) Blood flow dynamics map in control and acrylamide-treated (2.0 mM) zebrafish. (E to H) Relative mRNA expression of cardiac-development-related genes in control and acrylamide-treated zebrafish (*n* = 3 per group). (I) Experimental design: pregnant rat exposed to acrylamide (1, 5, and 10 mg/kg·bw per day) for 19 d. (J) PCA scores plots based on RNA-seq data of hearts from rat embryos exposed to acrylamide (10 mg/kg·bw per day) (*n* = 3 per group). (K) Cluster analysis and (L) volcano plot of differentially expressed genes based on RNA-seq analysis. (M) KEGG analysis of RNA-seq data. (N to V) Relative mRNA levels of cardiac-development-related genes in control and acrylamide-treated rat embryos (*n* = 4 per group). Data are presented as the means ± SEM. Significance was calculated using one-way ANOVA with Tukey’s post hoc test; groups labeled with different letters differed significantly (*P* < 0.05).

Our prior findings demonstrated how chronic exposure to acrylamide induces HF. We next reasoned that acrylamide might trigger abnormal cardiac development before the occurrence of HF but the mechanisms remain unclear. Subsequently, we developed models using embryonic zebrafish (AB strain) and pregnant rats (SD strain) to explore the potential toxicity of acrylamide on embryonic cardiac development during the early stages of life (Fig. 7). To visualize cardiovascular deformity, we treated the transgenic zebrafish expressing *Tg(myl7:GFP)* with acrylamide after 2 h postfertilization (hpf) and observed at 96 hpf when the heart functioned (Fig. 7A and B). We found that the embryonic hearts treated with acrylamide were morphologically stretched and elongated with little overlap between ventricle and atrium (Fig. 7B) and showed reduced heart rates, blood flow rates, and linear velocity (Fig. 7C and D, Fig. S11A and B, and Movie S2), indicating a deficient cardiovascular system. Moreover, we also investigated several key cardiac-specific transcription factors related to heart development at 5 dpf, including *Nkx2.5*, *Gata4*, *Tbx5a*, and *Atp2a*. The gene expression revealed that acrylamide exposure (2.0 mM) significantly elevates the expression of *Nkx2.5* (*P* < 0.05) and marginally up-regulates the expression of *Gata4* (Fig. 7E and F), while down-regulating the gene expression of *Tbx5a* and *Atp2a* with no significant change (Fig. 7G and H), evidencing the cardiotoxicity effect of acrylamide on ventricular maturation.

To continuously trace back to the toxic effect of acrylamide during the gestational period, we next sought to understand whether gestational exposure to acrylamide impairs the cardiac development of embryos (Fig. 7I). Embryos and their hearts were collected from pregnant rats at the 19th gestational day (GD 19). The RNA-seq analysis screened out 959 differential genes based on 3 biological replicates by comparing the acrylamide treatment (10 mg/kg·bw per day) group with the control group, including 424 up-regulated and 535 down-regulated genes (Fig. 7J to L). Interestingly, further KEGG analysis based on RNA-seq data enriched oxidative phosphorylation, cardiac muscle contraction, fatty acid degradation, dilated cardiomyopathy, arginine and proline metabolism, and hypertrophic cardiomyopathy pathways, which were closely correlated with cardiac development and significantly changed in acrylamide-exposed rat embryos (Fig. 7M). Subsequently, we evaluated the gene expression of heart development markers (*Bmp4*, *Lsl1*, *Gata4*, and *Hand1*) (Fig. 7N to Q), transcription factors (*Nkx2.5*, *Tbx5*, *Mef2c*, and *Fgf10*) (Fig. 7R to U), and extracellular-matrix-related gene (*Has2*) (Fig. 7V). Acrylamide significantly elevates the gene expression of *Nkx2.5* and *Tbx5* (*P* < 0.05), indicating the cardiotoxicity effect on ventricular maturation consistent with previous zebrafish results. Nevertheless, acrylamide exposure marginally up-regulates the expression of *Bmp4*, *Lsl1*, *Hand1*, and *Has2* and slightly down-regulates the expression of *Gata4*, *Mef2c*, and *Fgf10*. The expression of those disturbed genes leads to cardiac dysfunction during embryonic development, which further promotes the development and progression of HF later in life [38,39]. Therefore, exposure to acrylamide during pregnancy leads to the differential expression of genes associated with embryonic heart development, potentially influencing the occurrence of HF. Taken together, acrylamide induces abnormal cardiac development during early life stages in both zebrafish embryos and pregnant rats.

### The adverse acrylamide exposure threatens next generation via epigenetic change evoked by DNA methyltransferase 1 (DNMT1)

**Fig. 8.**
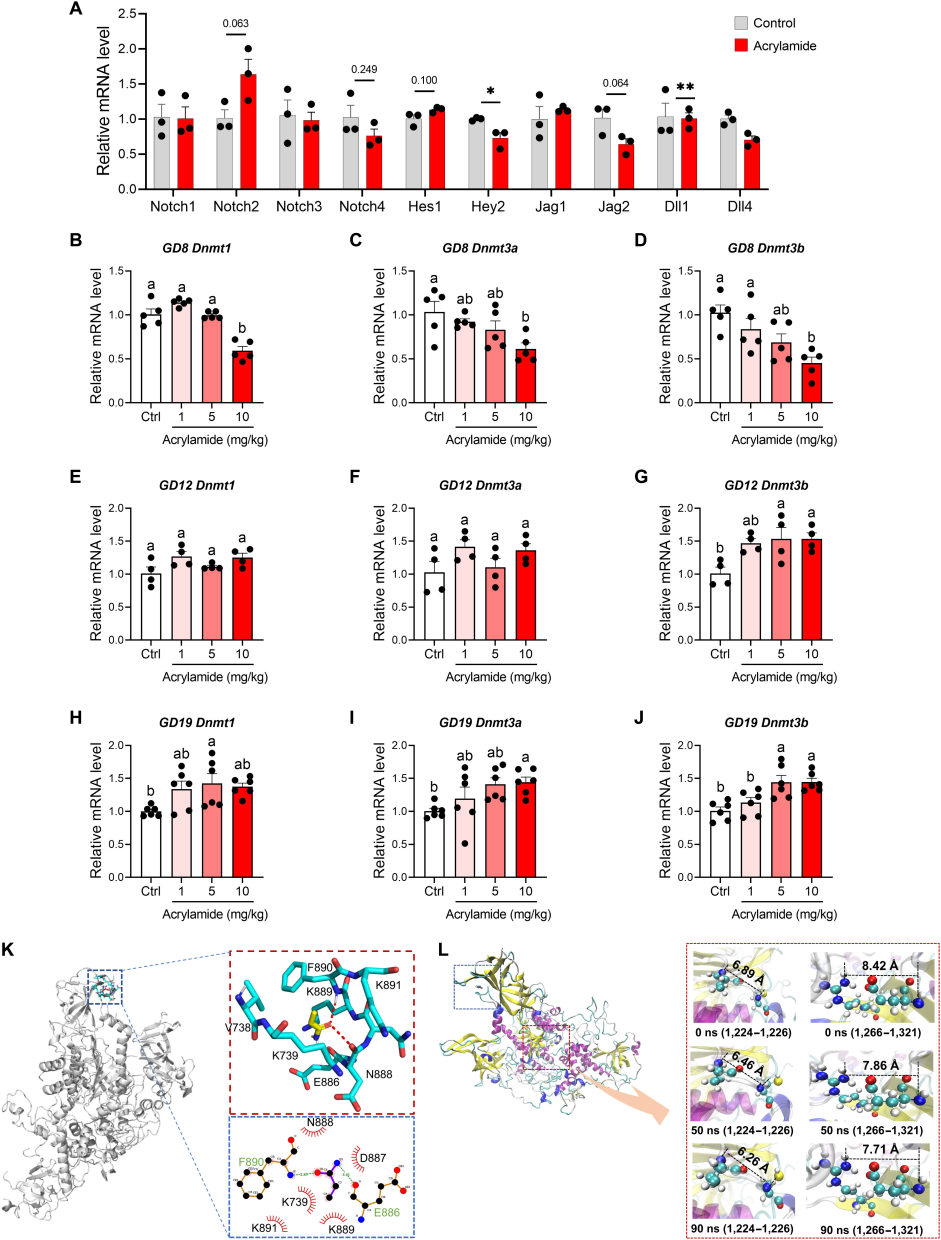
Acrylamide exposures generates epigenetic variations via DNA methylation. (A) Relative mRNA expression of cardiac NOTCH signaling pathway related genes in control and acrylamide-treated (10 mg/kg·bw per day) rat embryos (*n* = 3 per group). Relative mRNA expression of cardiac *Dnmt1*, *Dnmt3a*, and *Dnmt3b* genes in control and acrylamide-treated (1, 5, and 10 mg/kg·bw per day) rat embryos at (B to D) GD8 (*n* = 5 per group), (E to G) GD12 (*n* = 4 per group), and (H to J) GD19 (*n* = 6 per group). (K) Molecular docking results and residues of acrylamide interacting with DNMT1. (L) Acrylamide–DNMT1 conformational changes during the MD simulation: P1224 and C1226 are in the left, and E1266 and R1312 are in the right. Data are presented as the means ± SEM. Significance was calculated using 2-tailed *P* values by unpaired Student’s *t* test or one-way ANOVA with Tukey’s post hoc test; *P < 0.05 and **P < 0.01; groups labeled with different letters differed significantly (*P* < 0.05).

So far, acrylamide could malfunction heart development in zebrafish and rat embryos during early life stages, indicating the transgenerational toxic effects of parental exposure in offspring. To comprehensively address the epigenetic role of DNA methylation, we scrutinized the gene expression of DNA modification enzymes in hearts of adult zebrafish and mice (Fig. S11C and D). We found that chronic exposure to acrylamide suppresses the expression of *Dnmt8* in adult zebrafish heart and *Dnmt3a* in mouse heart, but with no significant changes (Fig. S11C and D). Then, we traced back to the expression of DNA modification enzymes during the early life stage. We found no noteworthy alterations in the expression levels of *Dnmt1*, *Dnmt3*, *Dnmt4*, *Dnmt5*, *Dnmt6*, *Dnmt7*, and *Dnmt8* with acrylamide exposure at 5 dpf (Fig. S11E to K). Notably, gestational exposure to acrylamide disturbs NOTCH pathway, especially in the ligand *Dll4* and the downstream signal *Hey2* in hearts of rat embryos (Fig. 8A), indicating that gestational exposure may alter DNA methylation associated with the occurrence of CVDs in rat embryos. Subsequently, we examined the expression of DNA modification enzymes in hearts of rat embryos at different gestational periods (GD8, GD12, and GD19). Interestingly, the expression of *Dnmt1*, *Dnmt3a*, and *Dnmt3b* decreases first and then increases in a dose-dependent manner during the progress of pregnancy with acrylamide exposure (Fig. 8B to J), indicating that acrylamide affects methylation modifications in early embryonic development [40]. These results demonstrate that acrylamide exhibits a potential epigenetic impact on the heart development and gestational exposure may induce aberrant cardiovascular disorders via DNA methylation modification. The maintenance enzyme DNMT1 plays a pivotal role in overseeing the majority of DNA methylation and ensuring its persistence throughout an organism’s lifetime [41]. To further understand how acrylamide binds to DNMT1, we used molecular docking and molecular dynamics (MD) simulations to elucidate the binding mechanism. The results of molecular docking analysis unveiled the formation of hydrogen bonds between acrylamide and specific residues of DNMT1 (Fig. 8K). Furthermore, the binding interactions were probed through the conventional MD simulations along the 100-ns MD trajectories (Fig. S11L and M). The root mean square deviation of DNMT1 was enhanced from 0.64 nm (apo–DNMT1) to 0.73 nm (acrylamide–DNMT1) (Fig. S11L), implying the destabilization of the DNMT1 conformation upon the binding of acrylamide. We analyzed snapshots from MD trajectories to investigate the overall structure and conformational changes within the binding domain of each docking complex at 0, 50, and 90 ns (Fig. 8L). The primary catalytic area for the transfer of methyl donors from *S*-adenosylhomocysteine to cytosine bases is the C-terminal catalytic domain of DNMT1, which is composed of residues P1224, C1226, E1266, and R1312 [42]. Notably, the relative positions of these 4 residues have undergone significant changes, as shown by the current snapshot. The distance between the functional groups of P1224 and C1226 in the acrylamide–DNMT1 complex began at 6.89 Å and decreased to 6.46 Å at 50 ns and 6.26 Å at 90 ns (Fig. 8L). This indicates that acrylamide causes conformational changes in the methyltransferase domain’s 3-dimensional structure, which reduces the catalytic activity of DNMT1. To elucidate the ligand–receptor interaction, we then calculated the binding energy *ΔG*_binding_ between acrylamide and DNMT1 as −20.423 ± 10.261 kJ/mol (Fig. S11N), indicating that acrylamide tightly binds to DNMT1 with a large polar solvation energy *ΔG*_polar_ (60.796 ± 7.363 kJ/mol) (Fig. S11N). *ΔE*_vdw_ was −49.179 ± 6.825 kJ/mol (Fig. S11N), and hydrogen bond interactions and hydrophobic interactions play crucial roles in the acrylamide–DNMT1 complex binding process. Thus, acrylamide has the ability to reposition its catalytic domain and enter into the binding pocket of DNMT1, disrupting the catalytic activity of DNMT1. Interestingly, long-term and low-dose exposure to acrylamide may interfere with the DNMT1-mediated DNA methylation state, indicating that the conformational remodeling of DNMT1 contributes to acrylamide-induced epigenetic toxicity. Taken together, when tracing back to the reason for acrylamide-induced HF, we recognize that frequent fried food consumption, a way to long-term dietary exposure to acrylamide, can also produce harmful epigenetic changes evoked by DNMT1 in offspring, thereby attracting public concerns about transgenerational inheritance risk of acrylamide-induced HF.

## Discussion

Fried foods, a beloved component of daily diets worldwide, unfortunately pose a significant contributing factor to the onset of various chronic diseases, including overweight/obesity [43], hypertension [43], CVD [44], type 2 diabetes [43,45], and anxiety/depression [46]. Previous study revealed fried food consumption in relation to higher risk of CVD and all-cause mortality [44]. However, the relationship between fried food consumption, especially fried potato consumption, and the incidence of HF, as well as the impact of dietary-sourced acrylamide, remained unclear until the current study. Our findings reveal a noteworthy association between frequent consumption of fried foods, particularly fried potatoes, and an increased risk of HF. This causal relationship can be attributed largely to the health hazards posed by acrylamide. This conclusion is supported by evidence demonstrating a positive association between hemoglobin adducts of acrylamide and the occurrence of HF in humans (Fig. 1). This serial population-based evidence drives our motivation to address how long-term exposure to acrylamide induces HF. In addition, acute exposure to acrylamide causes cardiac developmental damage and interferes with cardiomyocyte connections during ventricular morphogenesis in zebrafish embryos [36]. Unfortunately, only a limited number of studies have delved into the potential causation of acrylamide-triggered HF when administering with long-term and low-dose exposure.

HF is defined by a slow decline in cardiac function that leads to irregular heartbeat, edema, and eventually death. Here, we found that acrylamide induces cardiomyocyte damage, cardiac fibrosis, and severe bradycardia in zebrafish (Figs. 2B and 8B to D). Combined with expected findings from diagnosed biomarkers of HF in mice (Fig. 3C to J), we demonstrated acrylamide-induced HF at phenotypes. Cardiomyocyte loss through apoptosis and necroptosis, an important component in the pathogenesis of HF, is mediated by both death receptor and mitochondrial signaling [47]. Notably, apoptosis is considered to be a key pathophysiological process and the primary mediator leading to cell death during HF. The defining molecular event in apoptosis is the activation of caspases. Caspase activation takes place in complex IIa and complex IIb in the death receptor pathway and the apoptosome in the mitochondrial pathway. Apoptosome assembly is triggered by cytochrome C, which is released through permeabilization of the outer mitochondrial membrane [48]. Here, we showed acrylamide-induced cardiac apoptosis via up-regulating BAX and down-regulating BCL-2 expression (Fig. 6A and B). When the apoptotic protein BAX was released from binding to BCL-2, the cytochrome C would flow out into the cytoplasm and subsequently bind to pro-Caspase 9 to form apoptosomes, which further initiates downstream apoptosis and cell death program [49]. To expand our understanding, the metabolomics data regarding the identification of differential metabolites and energy metabolism pathways indicate the deficiency of adequate energy supply to the heart (Figs. 4F to I and 7A and Fig. S8). The majority of the energy needed for heart pumping activity is produced by mitochondria. Importantly, exposure to acrylamide leads to mitochondrial damage with ROS accumulation, cytochrome C outflow, and dropped mitochondrial membrane potential (Fig. 5D to M). Mitochondria are involved not only in essential biosynthetic and metabolic pathways but also in maintaining calcium and redox homeostasis and also act as key regulators of apoptosis [50]. Severe disruption of mitochondrial biogenesis and function, leading to excessively high levels of oxidative stress, is a defining feature of HF. The cellular toxicity of acrylamide derives from the imbalance between biological oxidants and antioxidants, leading to oxidative stress (Fig. 5D to G). Thus, excessive oxidation may lead to the destruction of macromolecules and ultimately accelerate cell death through apoptosis [51]. Accordingly, acrylamide-induced HF is related to mitochondrial dysfunction and the resulting apoptosis based on our metabolomics data regarding the differential metabolites and energy metabolism pathways.

NOTCH is a signaling protein that acts in mitochondria to maintain mitochondrial integrity in situations that may cause apoptotic cell death. It becomes activated through the cleavage of the ligand-bound receptor, leading to the generation of the active NOTCH intracellular domain [52]. Recent studies have also unveiled the involvement of activated NOTCH signaling in the antiapoptotic effects observed in rat cardiomyocytes [53]. Furthermore, the activation of NOTCH/HES1/AKT signaling contributes to cardioprotection against ischemia and reperfusion injury. Consistently, we proved that acrylamide significantly inhibits the expression of *Notch1*, *Hes1*, *Hey2*, and phosphorylated protein kinase B (p-AKT), contributing to the apoptosis (Figs. 2J, 3N, and 6F to J). NOTCH signaling acts on AKT and mitochondrial proteins mitofusion-1 (MFN1) and mitofusion-2 (MFN2), preventing BAX protein from damaging mitochondria and maintaining cell survival [54,55]. In addition, activating NOTCH pathway could reduce lipopolysaccharide-induced oxidative injury by regulating the expression of *Bcl-2*, *Bax*, and *cleaved Caspase 3* in H9c2 cells [56]. Notably, acrylamide activates the NOTCH signaling pathway at the early life stages, while suppressing the pathway with a long-term exposure (Fig. 6H). The early activation of NOTCH signaling is a stress repair response for acute exposure to acrylamide due to its antioxidant and antiapoptotic effects [37,54]. Unfortunately, the NOTCH signaling fails to maintain antioxidant and antiapoptotic effects in adult zebrafish and mice under chronic exposure to acrylamide [22,35]. Thus, NOTCH signaling inhibition leads to increased cardiomyocyte apoptosis and triggers eventual acrylamide-induced HF.

Prior research has confirmed the transmission of traits acquired through dietary intake, including fried foods, to offspring via gametes. It has also suggested the potential for persistent alteration of the genomic methylome as a form of epigenetic variation transmitted through gametes [57–60]. We currently demonstrate that parental environmental effects on offspring result from disruptions in DNA methylation transferase (Fig. 8). The compromised DNA methylation and subsequent epigenetic inheritance affect the expression of a specific set of paternally hypermethylated genes associated with myocardial apoptosis, thereby contributing to HF, including *Notch1* and downstream genes (*Hes1* and *Hey2*) (Fig. 8A). Long-term exposure to acrylamide induces the expression of DNMT in zebrafish and mice (Fig. S11C and D). Moreover, DNMT1 plays the central role in DNA methylation among the DNA methyltransferase family [61], while the catalytic activity of DNMT1 has been linked to changes in global differentially methylation levels [62]. From a structural point of view, we revealed that acrylamide leads to conformational changes of DNMT1 and thus disturbs the function in vivo (Fig. 8K and L) [42], suggesting the disruption of DNMT1 be the possible mechanism that acrylamide exhibits epigenetic toxicity, which was consistently supported by the studies on other exogenous compounds such as metformin, atenolol, and venlafaxine [63,64]. Interestingly, significant changes in acrylamide-induced DNA methylation are not revealed later in life, nor are they evident early in life, but significant changes during pregnancy determine the risk impact on offspring (Fig. 8B to J and Fig. S11C to K). In utero, acrylamide exposure during critical windows of myocardial cell development can affect epigenetic inheritance changes [65] associated with the development of HF in offspring [66]. In other words, exposure of pregnant females to acrylamide may induce epigenetic changes evoked by DNMT1 in the fetus, thereby influencing the development of HF later in life. Previous studies have demonstrated that parental diet and nutrition are key factors in determining the health of offspring [67]. Poor diet patterns, including high fat and high energy, malnutrition, and alcohol consumption, not only adversely affect individual health but also extent “damage” memory through epigenetic alterations to subgenerations. Given the substantial impact of diet and nutrition on epigenetic modifications, nutritional interventions are necessary for the transgenerational effects of poor diet patterns. Thus, continuous public health concerns and nutritional interventions are needed for transgenerational effect of acrylamide, which has profound implications for the health of human and our progeny.

Our study has some limitations that should be addressed in future research. First, because our study was not randomized, controlled, unmeasured, or residual confounding cannot be fully ruled out. Second, although large sample sizes from UK Biobank and NHANES were used in the current human study, further multiple population-based studies including different ethnic groups with various classes of ages would be more representative. In addition, longitudinal and longer-term follow-up studies would be necessary to better understand the strength and verify our conclusions. Third, despite the fact that the animal models have widely been used for HF study, they cannot be directly compared to human HF evaluation. Last, extrapolating results from molecular and cellular experiments to clinical implications faces many challenges. Further studies are also needed to evaluate the adverse effects of fried foods on HF in a population-based intervention trial.

**Fig. 9.**
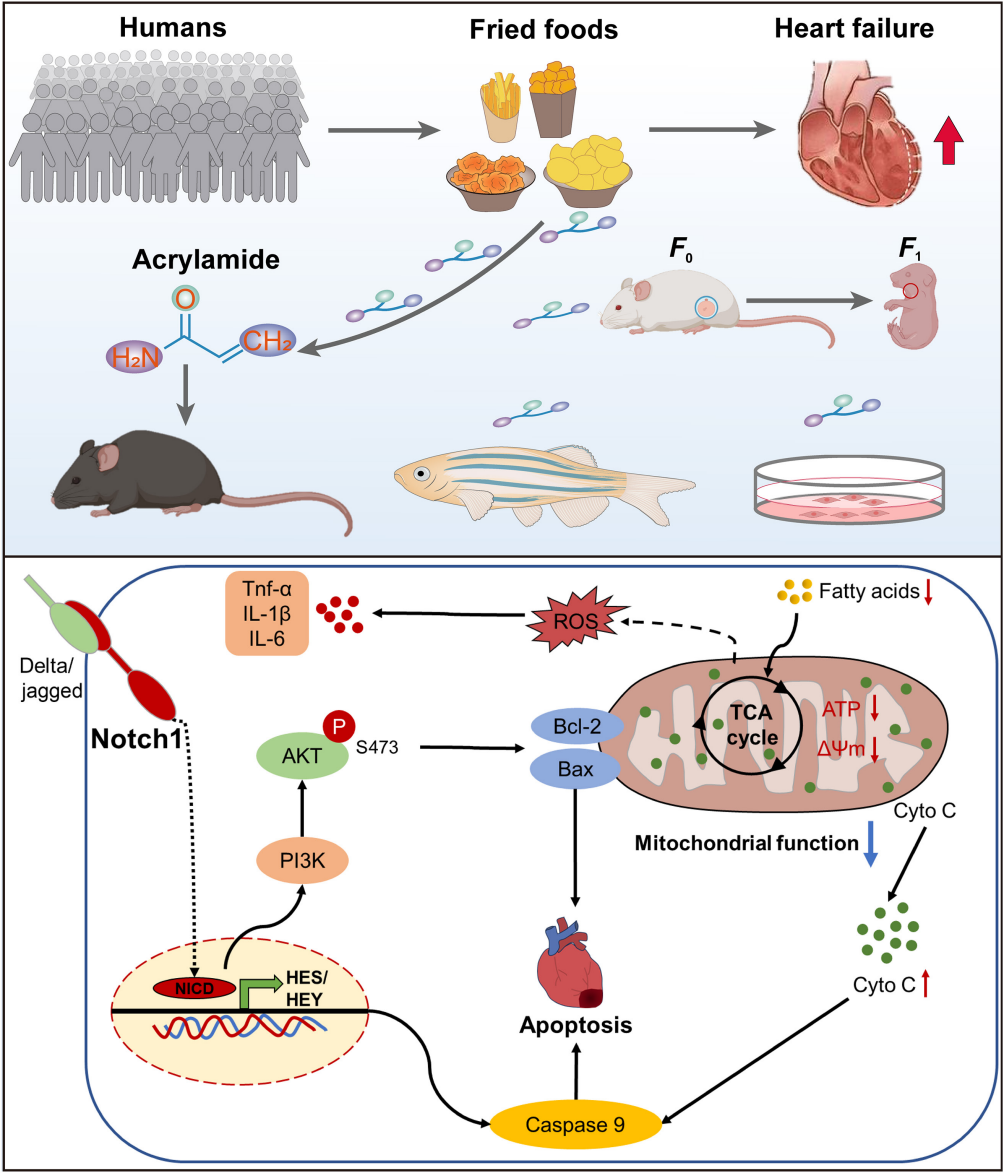
Main findings of this study. Briefly, this study demonstrates that frequent fried food consumption is strongly associated with higher HF risk due to the harmful effect of acrylamide in fried foods. The results reveal that long-term exposure to acrylamide induces HF through mitochondria disorder and NOTCH1-triggered apoptosis.

In conclusion, we reveal that frequent consumption of fried foods, identified as a dietary risk factor, significantly increases the risk of HF in humans. This heightened risk is primarily attributed to the production of acrylamide during the frying process, a correlation substantiated by population-based evidence linking hemoglobin biomarkers of acrylamide to a higher prevalence of HF. This study additionally illustrates that prolonged exposure to acrylamide induces HF in zebrafish and mice and elucidates the underlying mechanism, showing that chronic exposure to acrylamide disturbs cardiac energy metabolism due to the mitochondrial dysfunction and metabolic remodeling. Moreover, our findings support the notion that chronic acrylamide exposure induces myocardial apoptosis via inhibiting NOTCH1 signaling. Notably, acrylamide exposure during pregnancy epigenetically influences the development of HF in offspring. We recognize that frequent fried food consumption, a way to long-term dietary exposure to acrylamide, can also produce harmful epigenetic changes evoked by DNMT1 in offspring due to the transgenerational inheritance risk of acrylamide. Throughout the study, our results emphasize the crucial role of mitochondrial function and NOTCH1-triggered apoptosis in acrylamide-induced HF and contribute to a deeper understanding of the causal link and underlying mechanisms explaining the detrimental impact of fried food consumption on the transgenerational inheritance risk of HF (Fig. 9). In this regard, our study provides novel insights into the adverse effects of fried foods and acrylamide as a typical food processing contaminant on HF from population-based observations to experimental validation. These results indicate that more attention should be taken to the potential health risk caused by lifetime exposure to acrylamide in humans through fried food consumption.

## Materials and Methods

A total of 183,195 participants from the UK Biobank and 10,811 participants from the NHANES were eligible and included in the main analysis for the association of fried food consumption and acrylamide hemoglobin biomarkers with incident HF in our human study. The UK Biobank had ethical approval granted by the North West Multi-Centre Research Ethics Committee (reference number 06/MRE09/65), and all participants were required to provide written informed consent. Zebrafish, rat, mice, and cell models were used in this study to reveal the mechanisms of acrylamide exposure. Animal experiments were conducted according to the protocols approved by the Ethics Committee of Institutional Animal Care and Use Committee. For a detailed description of materials and methods, see Supplementary Detailed Methods.

## Data Availability

All data are available in the main text or the Supplementary Materials. Additional data related to this paper may be requested from the authors.

## Supplementary Materials

Supplementary 1 Detailed Methods Figs. S1 to S11 Tables S1 to S8 Movies S1 and S2
