# Supplementary material for: Habitual Daily Intake of Fried Foods Raises Transgenerational Inheritance Risk of Heart Failure Through NOTCH1-Triggered Apoptosis
Source: Research (Wash D C). 2024 Jul 15;7:0401. doi: 10.34133/research.0401 (PMC11246838; doi:10.34133/research.0401)
Supplement: Supplementary 1 — Detailed Methods Figs. S1 to S11 Tables S1 to S8 Movies S1 and S2 [file research.0401.f1.docx]

Supporting Information

Habitual daily intake of fried foods raises transgenerational inheritance risk of heart failure through Notch1-triggered apoptosis

**Detailed Methods**

**Supplementary Figures**

**Figure S1.** Flow diagram of the human study population in the UK Biobank.

**Figure S2.** Association of fried food, fried potato, and fried white meat consumption and the risk of HF stratified by potential risk factors.

**Figure S3.** Flow diagram of the human study population in the NHANES.

**Figure S4.** Chronic acrylamide exposure affected the global epigenetic changes in adult zebrafish hearts and induced the heart failure and inflammation response in adult mice hearts.

**Figure S5.** The statistical analysis of metabolomics in zebrafish hearts and mice hearts.

**Figure S6.** Chronic exposure to acrylamide induces hearts metabolic remodeling based on metabolomic analysis in zebrafish and mice.

**Figure S7.** The global KEGG network shows the hub metabolites in the global KEGG metabolic pathway in zebrafish and mice, respectively.

**Figure S8.** Chronic exposure to acrylamide induces hearts fatty acid metabolism disorder based on metabolomic analysis.

**Figure S9.** Chronic exposure to acrylamide may induce mitochondrial dysfunction and inhibit Notch1-PI3K/AKT signaling.

**Figure S10.** Acrylamide induces H9c2 cells death and disturbs Notch signaling pathway-related genes expression in H9c2 cells.

**Figure S11.** Acrylamide exposures generates epigenetic variations via DNA methylation.

**Supplementary Tables**

**Table S1.** Baseline characteristics of participants across fried food consumption in the UK biobank (*n*=183,195).

**Table S2.** Baseline characteristics of participants across fried potato consumption in the UK biobank (*n*=183,195).

**Table S3.** Baseline characteristics of participants across fried white meat consumption in the UK biobank (*n*=183,195).

**Table S4.** The associations between fried food, fried white meat, or fried potato consumption and heart failure risk (n=183,195).

**Table S5.** Multivariate-adjusted HRs (95% CIs) of heart failure according to fried food consumption from sensitivity analyses.

**Table S6.** Characteristics of study participants by quintiles of HbAA and HbGA in NHANES 2003-2006 and 2013-2016 (*n*=10,811).

**Table S7.** Multivariate-adjusted odds ratios (95% CIs) of the associations between acrylamide hemoglobin biomarkers and the prevalence of heart failure in NHANES 2003‒2006 and 2013‒2016.

**Table S8.** Primer pairs of selected genes in qRT-PCR analysis.

**Supplementary Videos**

**Video1** Video of mouse echocardiography.

**Video2** Video of zebrafish blood flow.

**Detailed Methods**

Ethics statement

The UK Biobank obtained ethical approval from the North West Multi-Centre Research Ethics Committee (reference number 06/MRE09/65), and all participants provided written informed consent. Animal experiments were conducted following approved protocols by the Ethics Committee of the Institutional Animal Care and Use Committee of Zhejiang University (Hangzhou, China) (Approval no. ZJU20210140 for zebrafish study) and Zhejiang Chinese Medical University (Hangzhou, China) (Approval no. IACUC‒20190429‒01 for rat study and IACUC‒20220425‒05 for mice study).

Human study

**UK Biobank study**

***Study design and participants in UK Biobank***

The UK Biobank is a nationwide prospective cohort that recruited over 500,000 participants aged 37 to 73 years from 2006 to 2010 [1]. All participants in this study had to be registered with a general practitioner and represent a wide range of exposures typical of the UK population [2]. The present study comprises 210,959 participants with dietary data via validated 24 h diet recall. After excluding individuals with CVD, cancer, and heart failure at baseline, as well as those who withdrew during the follow-up period, a final cohort of 183,195 participants was selected to assess the relationships of fried food consumption with HF risk (Figure S1, Supporting Information).

***Measurement of fried potato consumption***

A web-based questionnaire (The Oxford WebQ) aimed to record the consumption of over 200 common food and beverage items using a self-administrated 24‒h dietary recall approach, which has been validated against an interviewer-administrated 24-hour recall [3] and biomarkers [4]. The consumption of fried foods, fried white meat, and fried potatoes was estimated based on the first 24‒h dietary recall of each participant at baseline. The total consumption of fried food was determined by summing the intake of the following food items, including crumbed or deep-fried poultry, breaded or battered fish, fried potatoes or crisp, omelette, indian snacks, and doughnut. Among these, we defined crumbed or deep-fried poultry and breaded or battered fish as fried white meats. In this study, total fried food consumption was the primary exposure, while the most consumed types of fried food (fried white meats and fried potatoes) were the secondary exposure.

***Outcome measurement of heart failure***

The HF outcome was determined through a combination of self-reported information and hospital inpatient records. Data on hospital admissions and subsequent diagnoses of HF were acquired through records linkage to Health Episode Statistics in England and Wales, as well as the Scottish Morbidity Records in Scotland. HF cases were identified based on hospital admissions with the 10th Revision codes I11.0, I13.0, I13.2, I50.0, I50.1, and I50.9 from International Classification of Diseases [5].

***Measurements of covariates***

In this study, potential confounding variables were considered based on our understanding of the exposure-outcome association through discussions among co-authors and previous literature [6]. The confounding covariates included in this analysis were age, sex, race, body mass index (BMI), socioeconomic position, Townsend deprivation index, smoking status, alcohol consumption frequency, physical activity, and vegetable, fruit, whole grains, processed red meat, fish, sugar-sweetened beverages and total energy intake. Further information regarding these metrics is available on the UK Biobank's website (http: //www.ukbiobank.ac.uk).

**NHANES study**

***Study design and participants in NHANES***

In this study, we aggregated data on acrylamide hemoglobin biomarkers from four survey cycles, encompassing the years 2003–2004, 2005–2006, 2013–2014, and 2015–2016. Consequently, the NHANES dataset initially comprised 40,616 participants from 2003 to 2016. Among them, 19,108 participants were excluded to focus our analysis on the adult population aged 20 years or older. Participants without complete dietary information (n = 657) or those with missing hemoglobin biomarker data (n = 10,040) were also disqualified. Ultimately, 10,811 participants were taken into account for the present analysis (Figure S3, Supporting Information).

***Assessment of acrylamide hemoglobin biomarkers***

Fasting blood samples were collected from participants by nursing staff and stored at -20 °C until analysis. The levels of hemoglobin adducts of acrylamide (HbAA) and glycidamide (HbGA) were determined in whole blood or erythrocytes using the NHANES laboratory method [7].

***Ascertainment of heart failure***

HF case was defined based on self-reported personal interview data in the NHANES medical conditions questionnaire section (“Has a doctor ever told you that you had congestive heart failure?”) [8,9].

***Covariates***

In the current study, we meticulously considered potential confounding variables, encompassing demographic, socioeconomic, lifestyle, and dietary factors, to enhance the robustness of our analysis. Information on age, sex, race, BMI, education, family poverty-income ratio (PIR), physical activity, alcohol drinking status, total energy intake, and medical history was obtained through comprehensive household interviews. Additionally, smoking status was determined based on the level of serum cotinine (ng/mL). CVD cases just like HF were diagnosed through self-reports from participants. Through a series of 24-hour dietary recalls completed by skilled interviewers, daily dietary data for the NHANES were painstakingly gathered. The USDA Survey Nutrient Database was used to determine dietary nutrients and energy components.

Experimental animal study

**Zebrafish**

The wide-type AB strain and transgenic *Tg(cml2: EGFP)* of zebrafish (*Danio rerio*), which were provided by the Zebrafish Resource Center from China National Aquatic Biological Resource Center, were used in this study and were raised according to the husbandry guide from the *The Zebrafish Book* [10]. Healthy embryos were carefully selected under a stereo microscope (Olympus, Tokyo, Japan). Two hours post-fertilization (hpf), the viable embryos were transferred into 6-well plates and exposed to 6 ml of acrylamide (Sigma Aldrich; 0.5, 1.0, and 2.0 mM). The experimental solutions used were refreshed every day to ensure stable concentrations of acrylamide. The individual *Tg(cml2: EGFP)* zebrafish were carefully positioned using a spatula to ensure that the tail, body components, and eyes were all aligned in the same horizontal plane. Images were captured using a fluorescence microscope (Olympus, Tokyo, Japan).

For long-term exposure to acrylamide, 2 hpf healthy embryos were first exposed to acrylamide (Sigma Aldrich; 0.125 and 0.25 mM) in a Petri dish (100 embryos/50 ml EM) until reaching 5 dpf, with all embryos progressing to this stage. Subsequently, the fish were transferred to 2-L tanks at 5 dpf for a duration spanning from 5 to 30 dpf. During this period, the fish were provided with zebrafish larval diet three times between 5 and 14 dpf (Aquatic Habitats) and thereafter fed twice daily with freshly hatched live Artemia after 14 dpf. After 30 dpf, the fish were maintained in 10-L tanks (30 fish per tank) until the conclusion of the experiment. The exposure solution was renewed daily with a freshly prepared stock solution. After a 180‒day exposure to acrylamide, adult zebrafish were anaesthetized with tricaine (Sigma-Aldrich; final concentration 160 μg/ml). Heart samples (3 fish serving as 1 replicate per sex) were carefully collected from both the control and acrylamide-treated groups. Subsequently, the heart samples were then quickly frozen in liquid nitrogen and kept for further examination at -80 °C.

**Rats**

Two-month-old Sprague Dawley rats were provided by Shanghai Laboratory Animal Co., Ltd. (Shanghai, China) and kept in a rodent facility that had a fixed temperature of 24°C and a 12-hour light/dark cycle. The experimental female rats were randomly divided into 4 groups and orally administered with acrylamide (1, 5, and 10 mg/kg bw/day) or equivalent volumes of water as the control. After a 10-day adaptive exposure to acrylamide, a virgin female rat was mated with a male rat to induce pregnancy. We defined the day when the vaginal plug was found as the first gestational day (GD 0) [11]. Finally, on GD 19, before the birth of fetuses, the gestational rats were euthanized by carbon dioxide and carefully dissected to collect the embryos’ hearts. The samples were all rapidly frozen and then stored at ‒80°C for further analysis.

**Mice**

Eight weeks old C57BL/6J mice were purchased from Shanghai SLAC Laboratory Animal Co., Ltd. and housed in cages with two to five animals apiece. The mice were kept at a constant temperature of 23 °C and were subjected to a 12-hour light/dark cycle (lights on from 7:00 to 19:00). The mice had ad libitum access to food and water, and the relative humidity was maintained between 45–65%. They were provided with a standard rodent chow diet. The experimental male mice were randomly assigned to two groups and subjected to oral administration of either purified water or acrylamide (0.5 mg/kg body weight/day) for a duration of 90 days. Mice were euthanized by intraperitoneal injection of pentobarbital sodium (Sigma-Aldrich; 100 g/kg bw) and cervical dislocation was performed as ad secondary euthanasia procedure.

**Cells**

The National Collection of Authenticated Cell Cultures in Beijing, China is where the rat cardiomyoblast cell line, H9c2, was obtained. H9c2 cells were cultured in complete medium, which included 10% fetal bovine serum (Gibco, NY, USA) added to high-glucose Dulbecco's Modified Eagle's media (ATCC, MD, USA) and 1% antibiotics, which included 10 KU/mL penicillin and 10 mg/mL streptomycin (Meilunbio, Dalian, China). Cell cultures were kept at 37°C with 5% CO2 in a humidified incubator. Acrylamide (at concentrations of 10 and 100 μg/mL) was filtered through a sterile PES membrane (0.22 μm) twice before being dissolved in complete medium for cell treatment. Each cellular experiment was conducted independently at least three times.

**MTT assay of H9c2 cells**

After being seeded in 96-well plates, H9c2 cells were treated for 24 or 48 hours with either the entire medium or different doses of acrylamide (50, 100, 200, 400, and 800 μg/mL). The MTT assay with 3-(4,5-dimethylthiazol-2-yl)-2,5-diphenyltetrazolium bromide (Leagene, Beijing, China) was used to determine the vitality of the cells. Following a 4-hour incubation with MTT solution, the culture medium was aspirated, and dimethyl sulfoxide was added for 10 minutes to dissolve formazan crystals at the bottom. The absorbance was determined with a microplate reader at 490 nm in wavelength (Meigu, Shanghai, China).

**Detection of reactive oxygen species**

According to the manufacturer, the Reactive Oxygen Species Assay Kit (Beyotime, Shanghai, China) was used to quantify the amounts of reactive oxygen species (ROS) in H9c2 cells after they were exposed to acrylamide. Briefly, the 2',7'-dichlorodihydrofluorescein diacetate probe was used to load the cells, followed by washed two times with phosphate buffer saline (PBS), and dispersed into single-cell suspension with trypsin treatment. Fluorescence of each cell was collected by a CytoFLEX LX Flow Cytometer (Beckman Coulter, CA, USA), via channel of fluorescein isothiocyanate (FITC).

**Mitochondrial membrane potential assay**

The mitochondrial membrane potential was measured using the Mitochondrial Membrane Potential Assay Kit with JC-1 (Solarbio, Beijing, China), following the manufacturer's protocol. After trypsin treatment, H9c2 cells were dispersed into single−cell suspension, then loaded with the JC−1 probe. Fluorescence of each cell was collected by a CytoFLEX LX Flow Cytometer (Beckman Coulter, CA, USA), via channels of FITC and phycoerythrin (PE).

**Mitochondrial staining**

The Mito-Tracker Red CMXRos (Beyotime, Shanghai, China) was used to locate the mitochondria in H9c2 cells. In brief, glass cover slips were put into 24−well plates where cells were seeded. After acrylamide treatment, mitochondria in cells were incubated in complete medium with Mito-Tracker Red CMXRos for 40 min at 37°C and washed in PBS once for later Immunofluorescence staining.

**Immunofluorescence staining**

For immunofluorescence analysis, glass cover slips were put into 24−well plates with seeded cells. The cells were fixed with 4% paraformaldehyde (PFA) for 15 minutes at 37°C and permeabilized with 0.2% Triton X-100 for 10 minutes at room temperature. Subsequently, samples were blocked with 5% serum (corresponding to the origin of secondary antibodies) for an hour at room temperature. After that, cells were treated at room temperature for one hour with secondary antibodies. Finally, the coverslips were meticulously affixed to glass slides using DAPI-containing mounting solution (Beyotime, Shanghai, China). All images were taken with a TCS SP8 confocal microscope (Leica, Wetzlar, Germany). The primary antibodies used in this study included mouse anti-Cytochrome C (Abcam, Cambridge, UK), rabbit anti-His-Tag, and mouse anti-Myc-Tag (CST, MA, USA). Donkey anti-rabbit IgG-Alexa Fluor 488, donkey anti-mouse IgG-Alexa Fluor 568, and goat anti-mouse IgG-Alexa Fluor 488 were among the secondary antibodies (Thermo Fisher Scientific, MA, USA).

**Heart failure assessment of zebrafish**

Following aquatic exposure to acrylamide (2.0 mM), ten zebrafish embryos were randomly selected from both the control and acrylamide-exposed groups for visual observation. Resting zebrafish were recorded on video using the Zebrafish Blood Flow System (Viewpoint, Lyon, France). The blood flow videos were subsequently analyzed using ZebraBlood™ (v1.3.2, ViewPoint). This software detects changes in pixel density and integrates them with vessel diameter to calculate a flow rate in nL/s for each frame. Quantitative assessments were conducted through video-based analysis, and blood flow dynamics were subsequently evaluated.

**Echocardiography**

Transthoracic echocardiography for mice heart was conducted using a Visual Sonics Vevo770 Imaging System equipped with a 30 MHz high-frequency transducer following the method published previously [12].

**Histological staining**

The isolated hearts from both mice and zebrafish were perfused with a 4% paraformaldehyde/PBS solution through the aorta, subsequently harvested, and fixed in 4% paraformaldehyde for 24 hours at 4˚C. For paraffin sectioning, the fixed hearts underwent dehydration using an ethanol gradient, were cleared in xylene for 2 hours, and finally embedded in paraffin. The resulting sections (5 μm thickness) were stained using the Hematoxylin and Eosin Staining Kit (Yeasen Biotech) and the Masson’s Trichrome Kit (Yeasen Biotech) following the manufacturer's protocol.

**Serum brain natriuretic peptide (BNP) activity assay**

BNP assay was performed with the Mouse BNP ELISA Assay Kit (catalog number: D721185) from Sangon Biotech according to the manufacturer’s recommendation.

**Serum creatine kinase (CK) activity assay**

The CK assay was conducted using the Creatine Kinase Assay Kit (catalog number: A032-1-1) from Nanjing Jiancheng Bioengineering Institute, following the manufacturer's recommendations.

**Transcriptome analysis**

The hearts of rat embryos in acrylamide treatment group (10 mg/kg bw/day) and control group were sampled for transcriptome sequencing by Shanghai Genechem Co.,Ltd (Shanghai, China). We evaluated the RNA integrity number (RIN) with the Agilent 2100 bioanalyzer (Agilent, CA, USA). cDNA sequencing library was sequenced on a cBot Cluster Generation System using TruSeq PE Cluster Kit v3-cBot-HS (Illumia, CA, USA). Raw reads in fastq format underwent preprocessing to remove unqualified reads. Hisat2 v2.0.5 was employed to build an index of the reference genome. Fragment per kilobase of transcript per million mapped reads (FPKM) of each gene was calculated using featureCounts v1.5.0−p3. DESeq2 provided statistical routines for measuring differential expression in digital gene expression data based on the negative binomial distribution. The resulting *P*-values were adjusted using the Benjamini and Hochberg’s approach to control the false discovery rate. The criteria for identifying significant differential expression were set as an adjusted *P* value (*P*adj) < 0.05 and an absolute Log_2_FC (fold change) ≥ 1. Subsequently, the obtained DEGs were subjected to GO and KEGG functional annotation and enrichment analysis.

**Whole-Genome Bisulfite Sequencing (WGBS) analysis**

Bisulfite sequencing was employed to detect methylated genomic regions following acrylamide exposure. Genomic DNA (gDNA) was extracted from fresh adult zebrafish heart tissue from the control and acrylamide exposure groups. DNA purity, library construction, evaluation and sequencing, and differentially methylated regions (DMRs) were analyzed following the method published previously [13,14].

**Metabolomics analysis**

Fresh adult zebrafish heart and mice heart tissues were collected from acrylamide-exposed or control group. Metabolites were extracted, measured and analyzed following the method published previously [15].

**Quantitative real time PCR analysis**

Total RNA was isolated from tissue or cells using the TRIzol reagent (Thermo Fisher Scientific, Waltham, MA, USA) following the manufacturer's protocol. Subsequently, the extracted RNA was reverse transcribed to cDNA using the reverse transcription kit (Takara, Dalian, China). PCR amplification was performed with TB Premix Ex Taq II (Takara, Dalian, China), while the detection of real-time fluorescence was executed in a LightCycler® 480II-384 Instrument (Roche, Basel, Switzerland). The detection protocol included a pre-denaturation step at 95°C for 3 min, followed by 40 cycles of amplification at 95°C for 15s, 60°C for 30s, and 72°C for 30s, in accordance with the manufacturer’s instructions. Relative gene expression levels were quantified on the basis of the 2^-∆∆^*^C^*^t^ method. Primer sequences are available in Supplementary Table 8.

**Western blot analysis**

Radioimmunoprecipitation assay (RIPA) lysis buffer from Solarbio, Beijing, China, was utilized to extract total proteins from H9c2 cells and mouse hearts, with a mixture of RIPA lysis buffer, phenylmethylsulfonyl fluoride, and phosphatase inhibitor at a ratio of 97:1:2, respectively. Subsequently, protein samples underwent separation through dodecyl sulfate polyacrylamide gel electrophoresis (6% for concentration and 10% for separation), followed by transferred to PVDF membranes. Blocking of membranes occurred in 5% nonfat milk, followed by overnight incubation with primary antibodies at 4 °C and subsequent treatment with horseradish peroxidase-conjugated secondary antibodies for 1 hour based on the target protein. Tris-buffered saline with 0.1% Tween-20 was used for membrane washing between steps. Finally, the bands were visualized by the enhanced chemiluminescence (Clinx, Shanghai, China). The relative expressions of proteins were analyzed by ImageJ software with GAPDH as internal control. Primary antibodies included rabbit anti-BCL−2 (Affinity, Jiangsu, China), rabbit anti-Cleaved Caspase 9 (Affinity, Jiangsu, China), rabbit anti-Pro-Caspase 9 (Affinity, Jiangsu, China), rabbit anti-BAX (Affinity, Jiangsu, China), rabbit anti-AKT (Affinity, Jiangsu, China), rabbit anti-S473 p-AKT (Affinity, Jiangsu, China), rabbit anti-DLL1 (Affinity, Jiangsu, China), rabbit anti-NOTCH1 (Abcam, Cambridge, UK), rabbit anti-GAPDH (Earthox, CA, USA). The secondary antibody, goat-anti-rabbit IgG, was obtained from Earthox company (CA, USA).

**Molecular Docking**

The crystal structure of DNMT1 (PDB: 4WXX, 2.62 Å) utilized in this study was obtained from the Protein Data Bank (PDB) structure database (<http://www.rcsb.org/pdb>). The structure of acrylamide for molecular docking calculations was sourced from the PubChem database (<http://www.ncbi.nlm.nih.gov/pccompound>). Molecular docking of acrylamide and DNMT1 were performed using Autodock following the method published previously [16]. The best conformation was chosen according to the lowest binding score for analysis.

**Molecular Dynamics Simulation**

MD simulation of the acrylamide-DNMT1 complex were performed using GROMACS 5.1.5 program package. The optimal orientations of the acrylamide-DNMT1 complex, obtained from molecular docking studies, served as the starting point for the MD simulation. The compound's center of mass was positioned at the center of a cubic box. The topology file for DNMT1 was generated using the CHARMM27 full atomic force field. Subsequently, the acrylamide-DNMT1 complex underwent minimization, equilibration to 300 K, and continuous data collection for 100 ns. The molecular mechanics Poisson-Boltzmann surface area (MM/PBSA) method was employed to calculate the compound's binding free energy.

Statistical analysis

In this study based on the UK Biobank, we summarized variables as means, standard deviations, or percentages. To assess the risk of HF, Cox proportional hazards regression models were employed, categorizing individuals into three levels based on their consumption of fried food, fried white meat, and fried potatoes. We adopted a three-step modeling approach for HRs and 95% CIs. A three-step modeling approach was implemented to calculate HRs and CIs for HF. Model 1 was adjusted for age and sex. Model 2 included additional adjustments for race/ethnicity, BMI categories, household income, Townsend deprivation index, smoking habits, alcohol consumption, and physical activity. Finally, model 3 further adjusted for dietary factors, encompassing vegetable intake, fruit intake, whole grains intake, processed red meat intake, fish intake, sugar-sweetened beverages intake, and total energy intake based on model 2. In addition, we investigated potential variations in the associations between fried food and fried potato consumption with HF risk across different subgroups stratified by sex, age, BMI, household income, Townsend deprivation index, smoking status, physical activity, alcohol consumption, and energy intake. The likelihood ratio test was used to assess the *P* value for interaction. Sensitivity analyses were conducted to test the robustness of our findings, considering adjustments for participants with medical history (T2D and CVD), vitamin and mineral supplementation, medication use, excluding incident heart failure within 5 years, or excluding participants with missing covariate data (Supplementary Table 5). Statistical significance was identified with a two-sided *P* value less than 0.05, and all analyses were performed using SAS version 9.4 (SAS Institute Inc., Cary, NC).

In this NHANES study, we accounted for the complex multistage probability sampling strategy inherent to NHANES by incorporating the sampling weights, strata, and primary sampling units provided by the National Center for Health Statistics (NCHS) into all statistical analyses. Due to skewedness, the levels of HbAA and HbGA underwent natural log-transformation. Categorical analyses were performed to examine the relationships between hemoglobin adducts of acrylamide and the prevalence of HF using multivariate-adjusted logistic regression models across quartiles. We employed a three-step modeling approach to compute the ORs and CIs for HF prevalence. Model 1 was adjusted for age, sex, and race. Model 2 extended the adjustments to include BMI, education, PIR, physical activity, smoking, drinking status, and mutually log-transformed HbAA (for HbGA) and HbGA (for HbAA). Finally, model 3 was adjusted for total energy intake based on model 2. All statistical analyses were performed using SAS version 9.4 (SAS Institute Inc., Cary, NC), and statistical significance was defined as a two-sided *P* value less than 0.05.

For animal and cell study, all the data in the test were demonstrated as the mean ± standard error of mean (SEM). Statistical analyses were performed using GraphPad Prism software. Group comparisons utilized the two-tailed Student's t-test (for pairwise comparisons) or one-way ANOVA with Tukey's post hoc test (for comparisons involving multiple groups). Statistical significance was determined as differences with a *P* value < 0.05.

**References**

1. Sudlow C, Gallacher J, Allen N, Beral V, Burton P, Danesh J, et al. UK biobank: an open access resource for identifying the causes of a wide range of complex diseases of middle and old age. *PLoS Med.* 2015;12(3):e1001779.

2. Hao G, Zuo L, Xiong P, Chen L, Liang X, Jing C. Associations of PM2.5 and road traffic noise with mental health: Evidence from UK Biobank. *Environ Res.* 2022;207:112221.

3. Liu B, Young H, Crowe FL, Benson VS, Spencer EA, Key TJ, et al. Development and evaluation of the Oxford WebQ, a low-cost, web-based method for assessment of previous 24 h dietary intakes in large-scale prospective studies. *Public Health Nutr.* 2011;14(11):1998-2005.

4. Greenwood DC, Hardie LJ, Frost GS, Alwan NA, Bradbury KE, Carter M, et al. Validation of the Oxford WebQ Online 24-Hour Dietary Questionnaire Using Biomarkers. *Am J Epidemiol.* 2019;188(10):1858-1867.

5. Algorithmically-defined health outcomes: UK Biobank. https://biobank.ndph.ox.ac.uk/showcase/label.cgi?id=42.

6. Kandola AA, Del Pozo Cruz B, Osborn DPJ, Stubbs B, Choi KW, Hayes JF. Impact of replacing sedentary behaviour with other movement behaviours on depression and anxiety symptoms: a prospective cohort study in the UK Biobank. *BMC Med.* 2021;19(1):133.

7. Pfeiffer CM, Sternberg MR, Schleicher RL, Haynes BMH, Rybak ME, Pirkle JL. The CDC's Second National Report on Biochemical Indicators of Diet and Nutrition in the U.S. Population Is a Valuable Tool for Researchers and Policy Makers. *J Nutr.* 2013;143(6):938S-947S.

8. Sattler ELP, Ishikawa Y, Trivedi-Kapoor R, Zhang D, Quyyumi AA, Dunbar SB. Association between the Prognostic Nutritional Index and Dietary Intake in Community-Dwelling Older Adults with Heart Failure: Findings from NHANES III. *Nutrients.* 2019;11(11):2608.

9. Yan Y, Mao M, Li Y-Q, Chen Y-J, Yu H-D, Xie W-Z, et al. Periodontitis Is Associated With Heart Failure: A Population-Based Study (NHANES III). *Front Physiol.* 2022;13:854606.

10. Westerfield, M. The Zebrafish Book: a Guide for the Laboratory Use of Zebrafish (Brachydanio) rerio. Eugene, USA:University of Oregon Press; 1993.

11. Domoradzki JY, Pottenger LH, Thornton CM, Hansen SC, Card TL, Markham DA, et al. Metabolism and pharmacokinetics of bisphenol A (BPA) and the embryo-fetal distribution of BPA and BPA-monoglucuronide in CD Sprague-Dawley rats at three gestational stages. *Toxicol Sci.* 2003;76(1):21-34.

12. Yu H, Zhang F, Yan P, Zhang S, Lou Y, Geng Z, et al. LARP7 Protects Against Heart Failure by Enhancing Mitochondrial Biogenesis. *Circulation*. 2021;143(20):2007-2022.

13. Jiang J, Li X, Yang K, Wang Y, Ye M, Wang W, et al. Formate for enhancing the growth of microalgae and accumulating high-value products. *Algal Research*. 2023;75:103261.

14. Shi C, Zhang J, Yan Z, Gao L, Gao C, Wu W, et al. Epigenetic effect of putrescine supplementation during in vitro maturation of oocytes on offspring in mice. *J Assist Reprod Genet.* 2022;39(3):681-694.

15. Wang A, Chen X, Wu S, Jia W, Jiao J, Zhang Y. Unraveling the Serum Metabolomic Profile of Acrylamide-Induced Cardiovascular Toxicity. *J Agric Food Chem*. 2021;69(40):12012-12020.

16. Lin W, Yan Y, Ping S, Li P, Li D, Hu J, et al. Metformin-Induced Epigenetic Toxicity in Zebrafish: Experimental and Molecular Dynamics Simulation Studies. *Environ. Sci. Technol.* 2021; 55(3): 1672–1681.

**
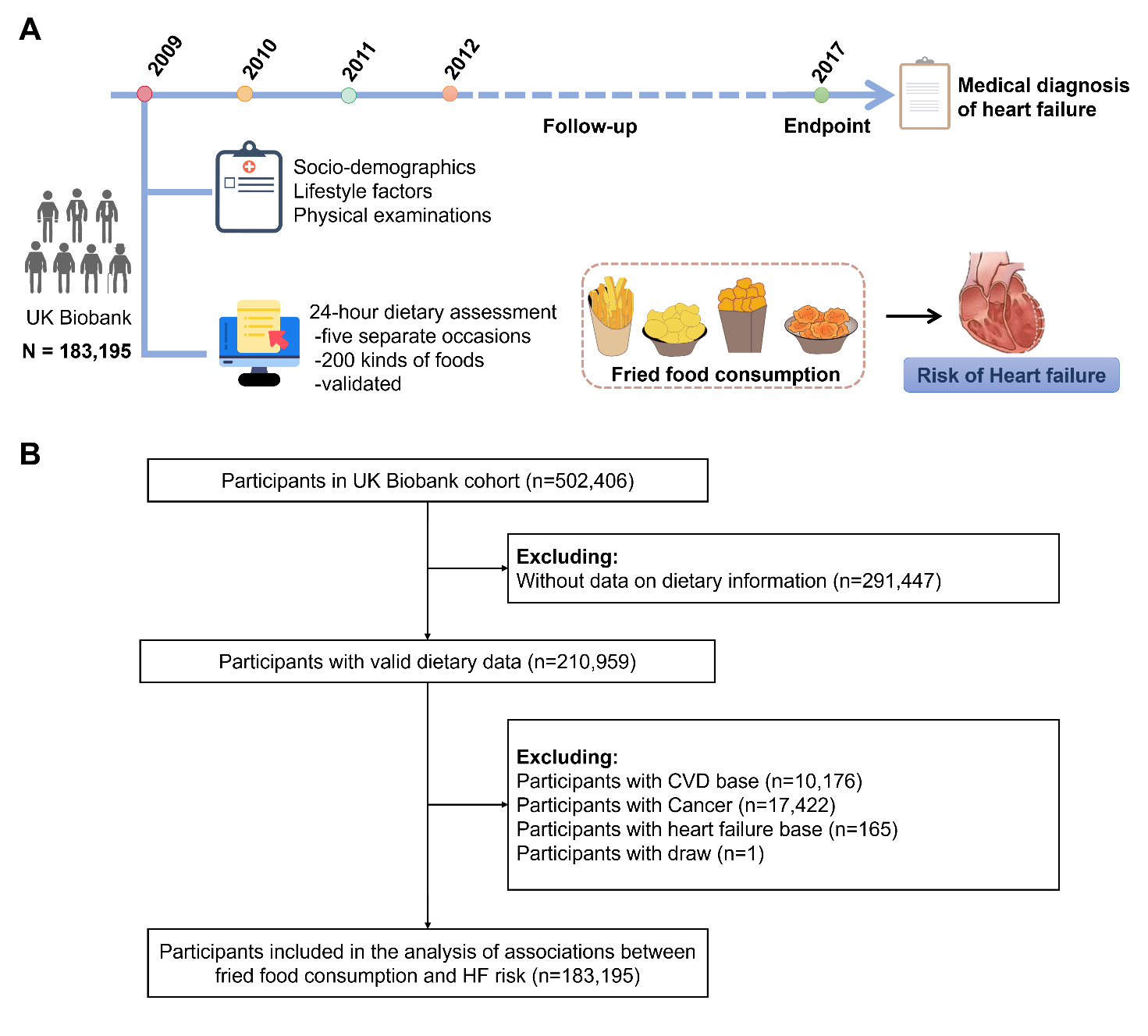
**

**Figure S1. Flow diagram of the human study population in the UK Biobank.**

**Figure S2.** **Association of fried food, fried potato, and fried white meat consumption and the risk of HF stratified by potential risk factors.** HRs were adjusted for age, sex, income, race center, BMI, TDI, smoking, alcohol use, physical activity, vegetable intake, fruit intake, whole grains intake, processed red meat intake, fish intake, sugar sweetened beverages intake, total energy intake (quartiles).

**
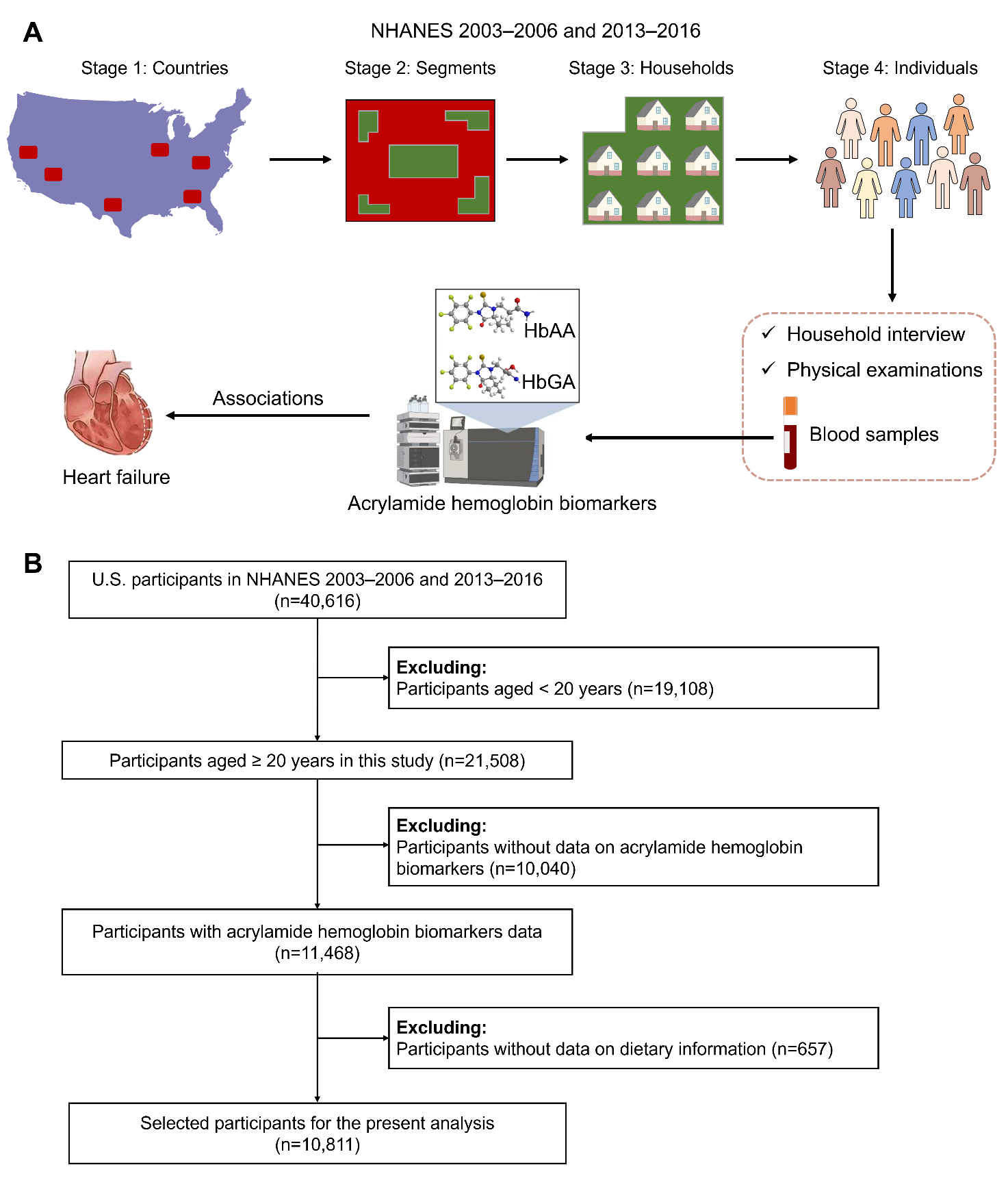
**

**Figure S3.** **Flow diagram of the human study population in the NHANES.**

**
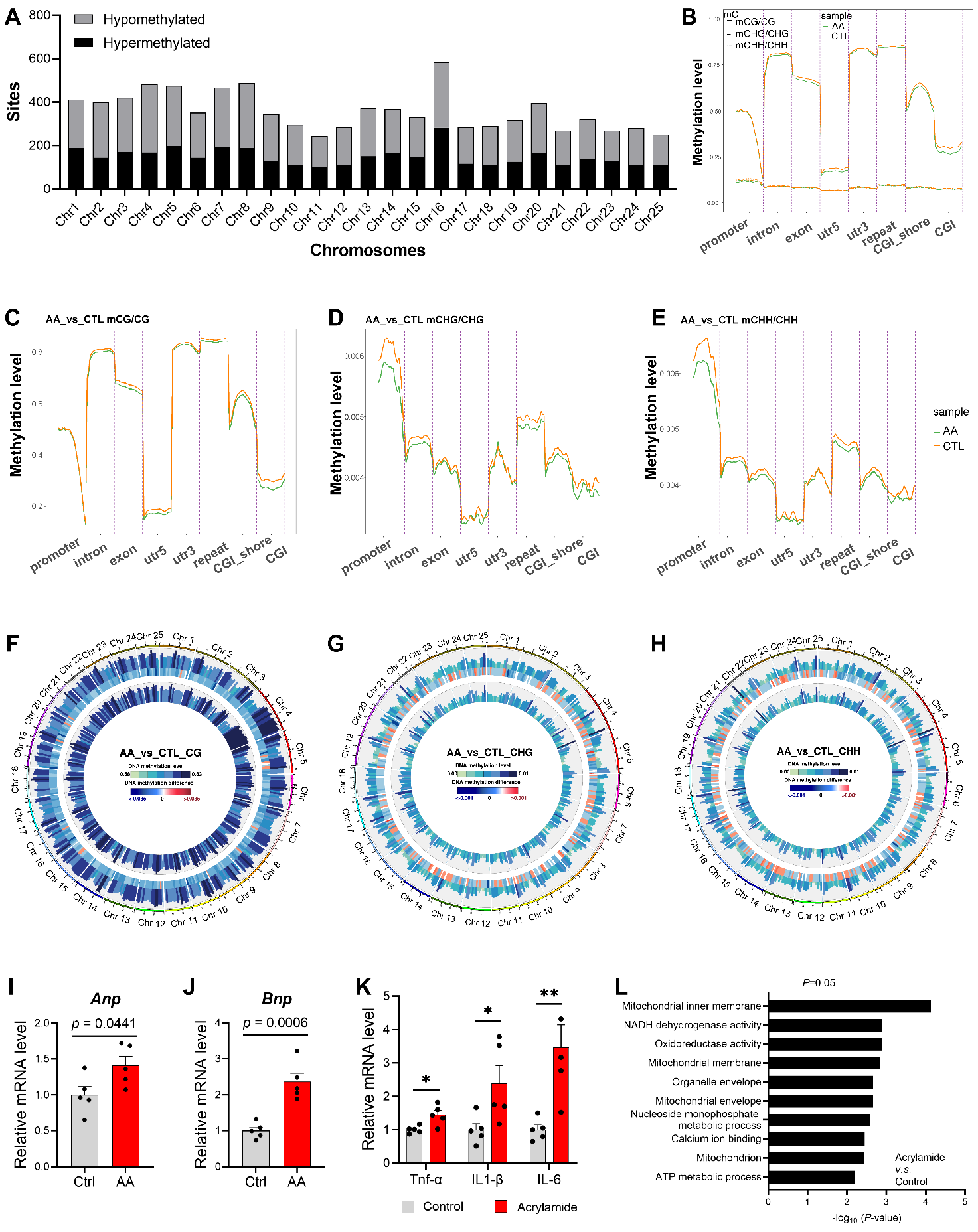
**

**Figure S4.** **Chronic acrylamide exposure affected the global epigenetic changes in adult zebrafish hearts and induced the heart failure and inflammation response in adult mice hearts.** (A) Hypermethylation and hypomethylation per chromosome in adult zebrafish hearts exposed to 0.25 mM acrylamide from 2 hpf to 180 dpf. Percentage of hypermethylated sites per chromosome are shaded black and percentage of hypomethylated regions is gray. (B) Global methylation level and methylation (C) at CG site, (D) CHG site, and (E) CHH site, respectively. The abscissa represents different genome elements, the ordinate represents methylation levels, and the different colors represent groups. (F‒H) Methylation patterns in zebrafish hearts exposed to 0.25 mM acrylamide at CG site, CHG site, and CHH site, respectively. (I and J) Relative mRNA levels of cardiac *Anp* and *Bnp* in control and acrylamide treatment groups (*n*=5 per group). (K) Relative mRNA levels of cardiac inflammation response in control and acrylamide treatment groups (n=5 per group). (L) GO analysis of RNA-seq data from hearts of rat embryos. Data are presented as the mean ± SEM. Significance was calculated using two-tailed *P* values by unpaired Student’s *t*-test; **P* < 0.05, ***P* < 0.01, and ****P* < 0.001.

**
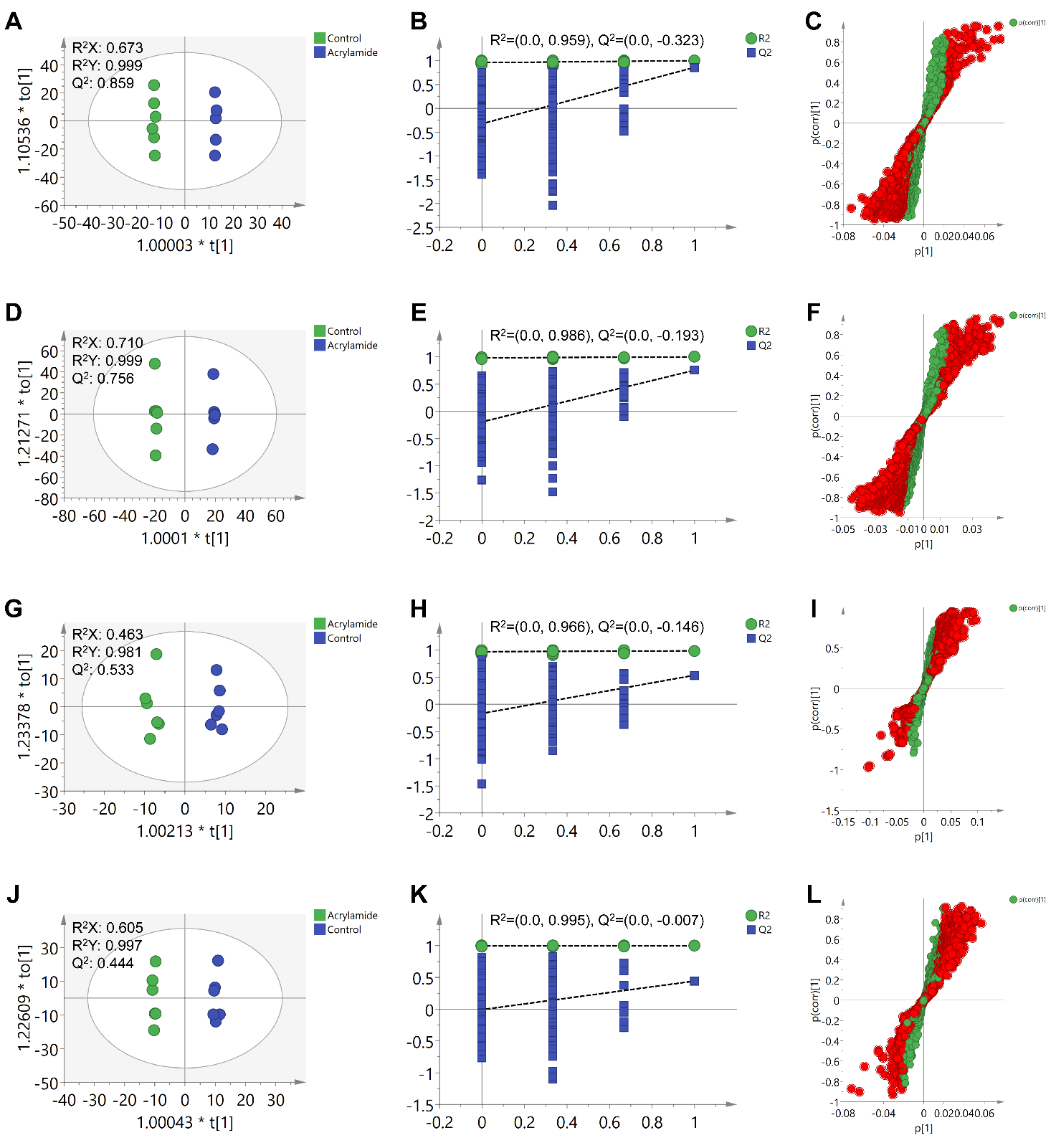
**

**Figure S5.** **The statistical analysis of metabolomics in zebrafish hearts and mice hearts.** (A and D) OPLS-DA scores plots, (B and E) statistical validation of the corresponding OPLS-DA model by permutation analysis (200 times), and (C and F) S-plot models derived from the UHPLC-MS/MS analysis for the zebrafish hearts with chronic acrylamide exposure (0.25 mM) by positive and negative modes, respectively. (G and J) OPLS-DA scores plots, (H and K) statistical validation of the corresponding OPLS-DA model by permutation analysis (200 times), and (I and L) S-plot models derived from the UHPLC-MS/MS analysis for the mice hearts with chronic acrylamide exposure (1 mg/kg·bw/day) by positive and negative modes, respectively.

**
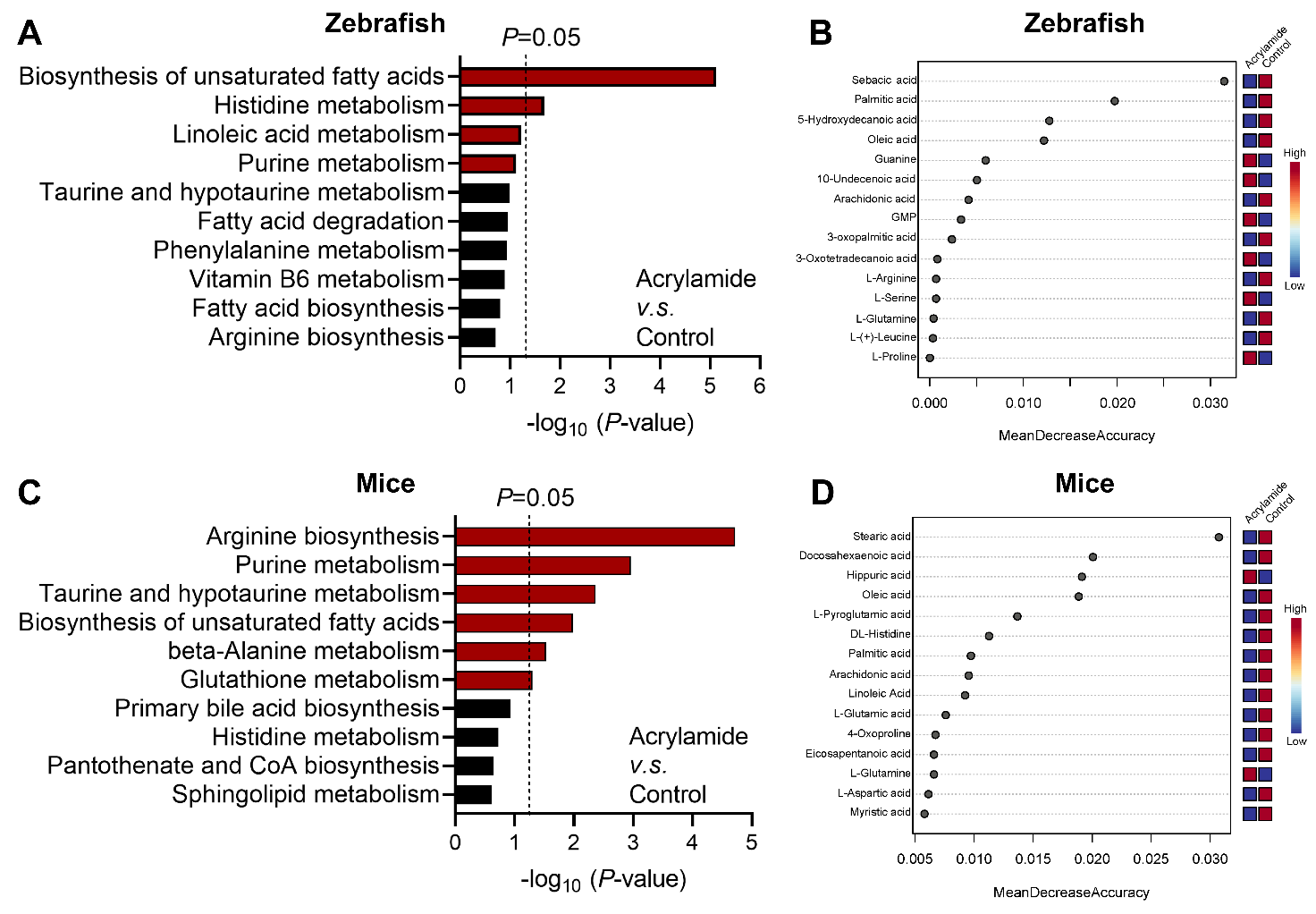
**

**Figure S6.** **Chronic exposure to acrylamide induces hearts metabolic remodeling based on metabolomic analysis in zebrafish and mice.** (A and C) KEGG pathway analysis of differential metabolites in zebrafish hearts and mice hearts, respectively. (B and D) Random forest of differential metabolites; features ranked by their contributions to classification accuracy (Mean Decrease Accuracy) in zebrafish and mice, respectively.

**
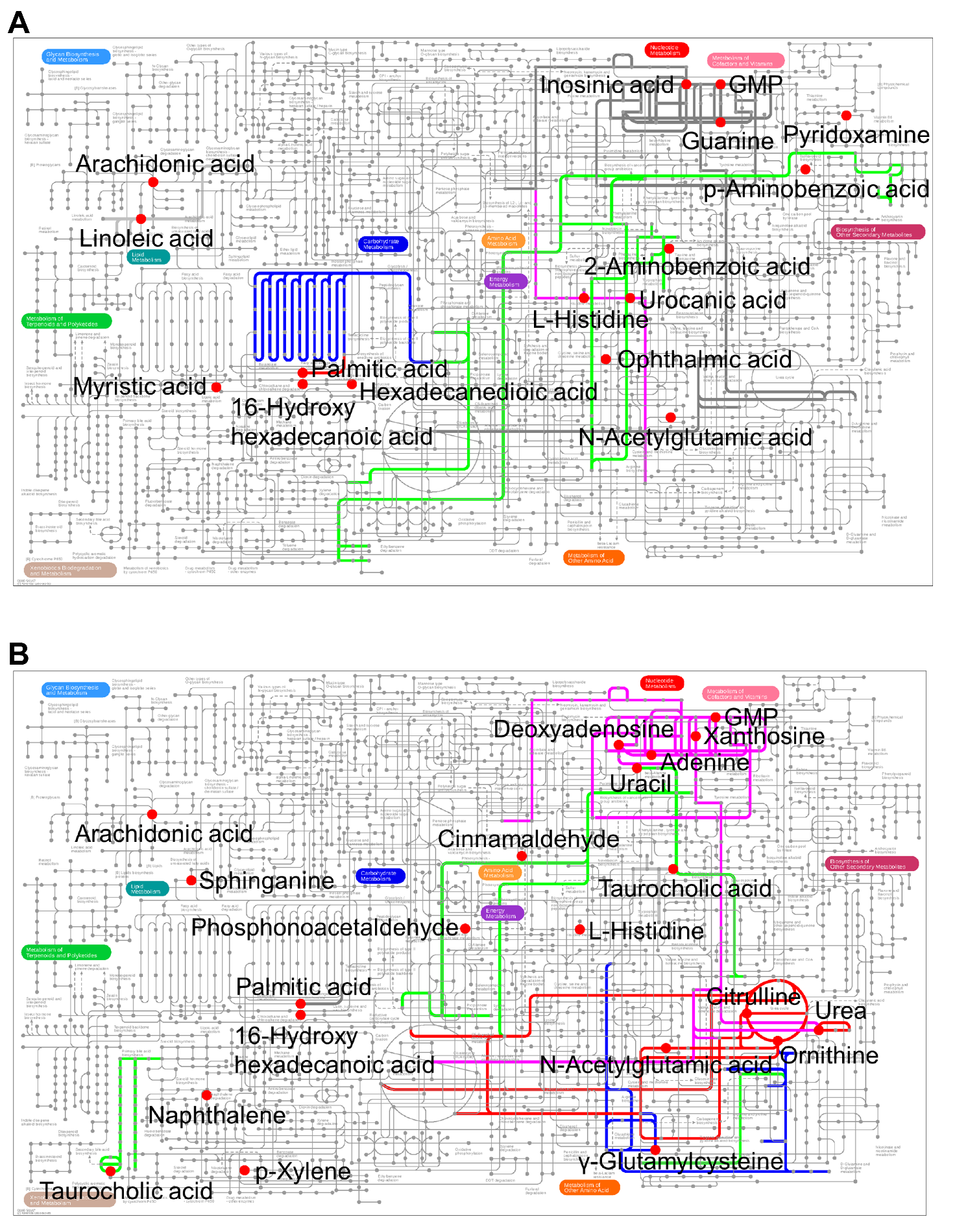
**

**Figure S7.** **The global KEGG network shows the hub metabolites in the global KEGG metabolic pathway in (A) zebrafish and (B) mice, respectively.**

**
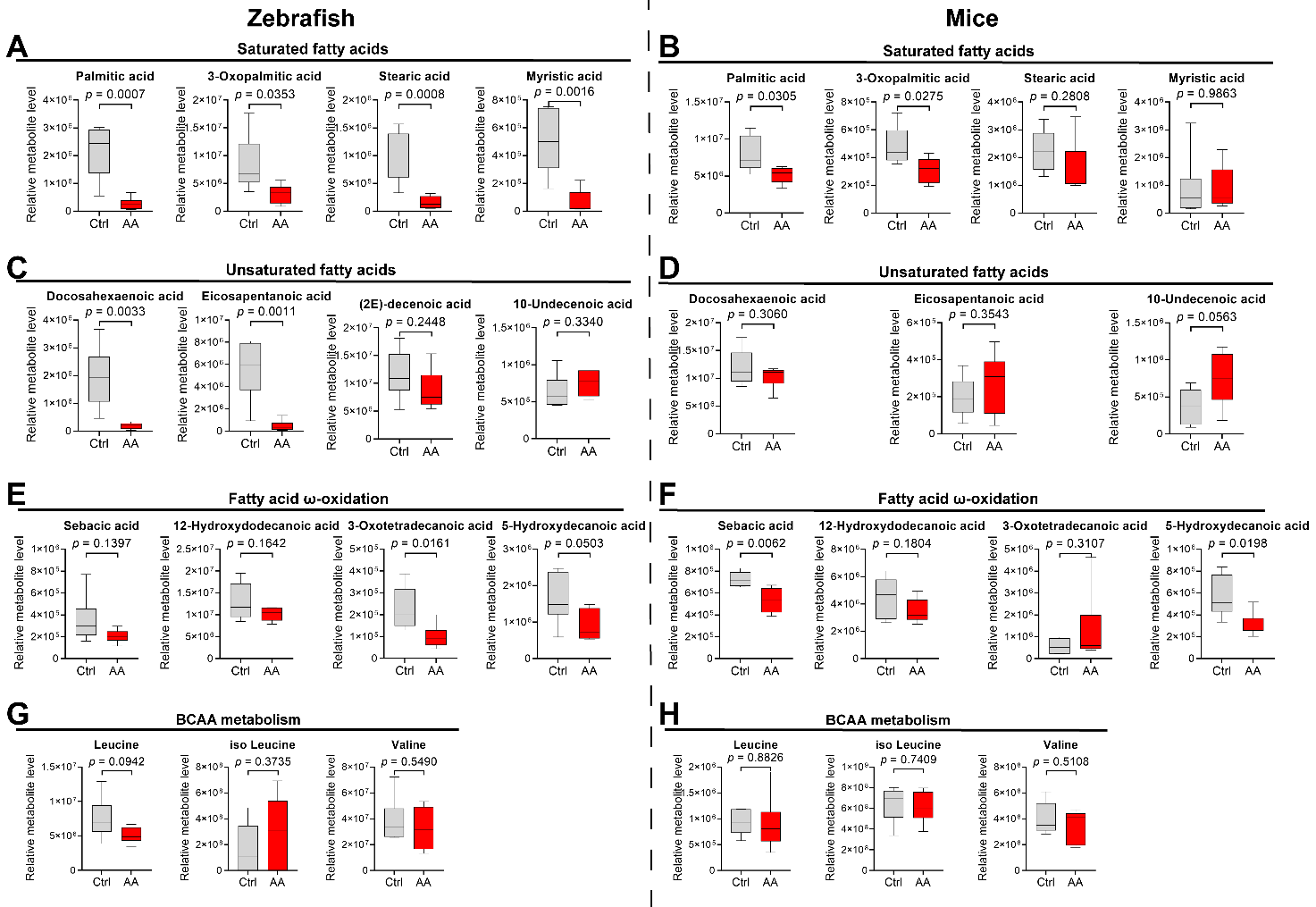
**

**Figure S8.** **Chronic exposure to acrylamide induces hearts fatty acid metabolism disorder based on metabolomic analysis.** (A and B) The relative saturated fatty acids levels in zebrafish hearts and mice hearts, respectively. (C and D) The relative unsaturated fatty acids levels in zebrafish hearts and mice hearts, respectively. (E and F) The relative fatty acid ω-oxidation levels in zebrafish hearts and mice hearts, respectively. (G and H) The relative BCAA metabolism levels in zebrafish hearts and mice hearts, respectively. Data are presented as the mean ± SEM. Significance was calculated using two-tailed *P* values by unpaired Student’s *t*-test.


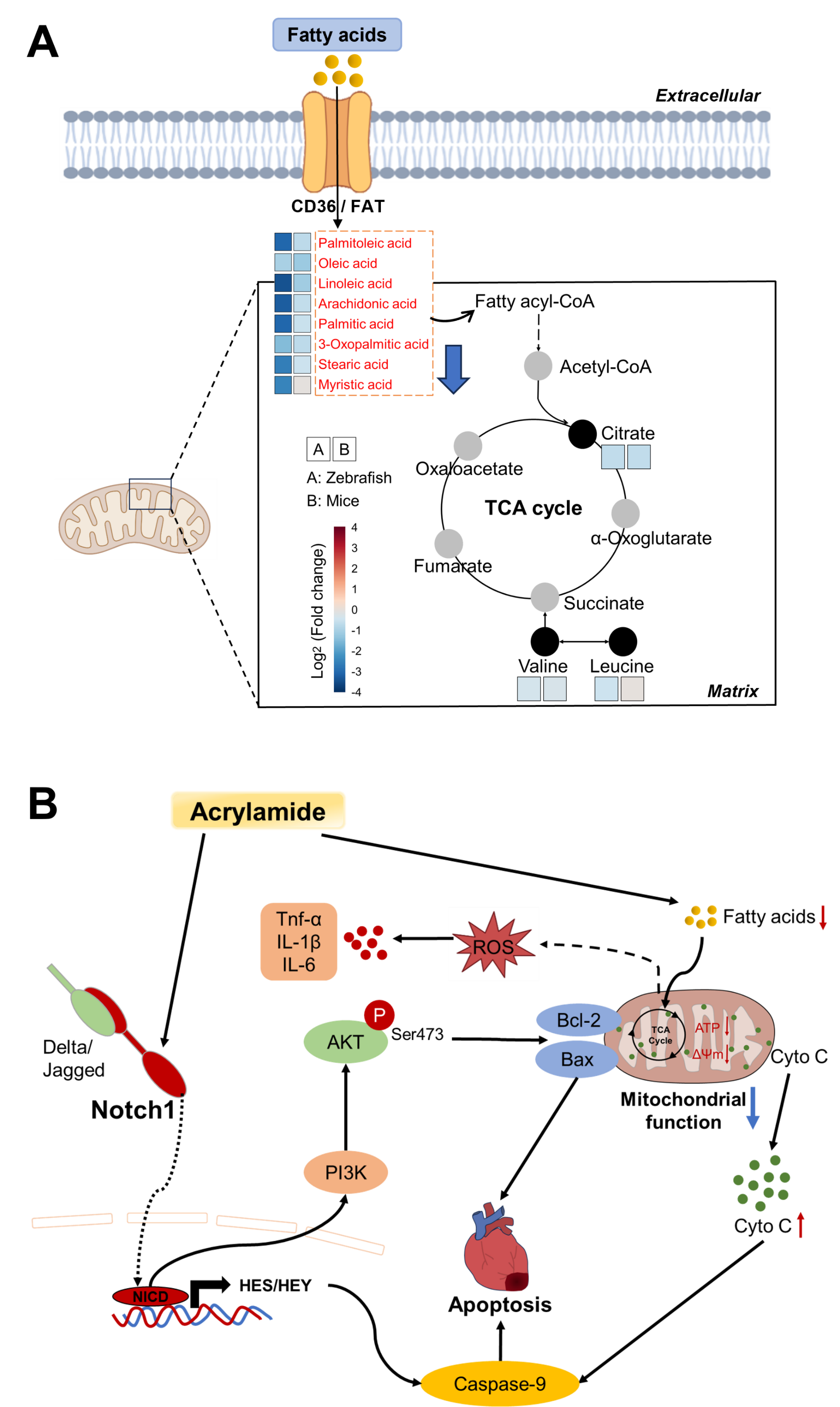


**Figure S9. Chronic exposure to acrylamide may induce mitochondrial dysfunction and inhibit Notch1-PI3K/AKT signaling.** (A) The metabolic network shows the reduction of mitochondrial fatty acid oxidation in zebrafish and mice. (B) The key role of mitochondrial function and Notch1-triggered apoptosis in acrylamide-induced HF.

**
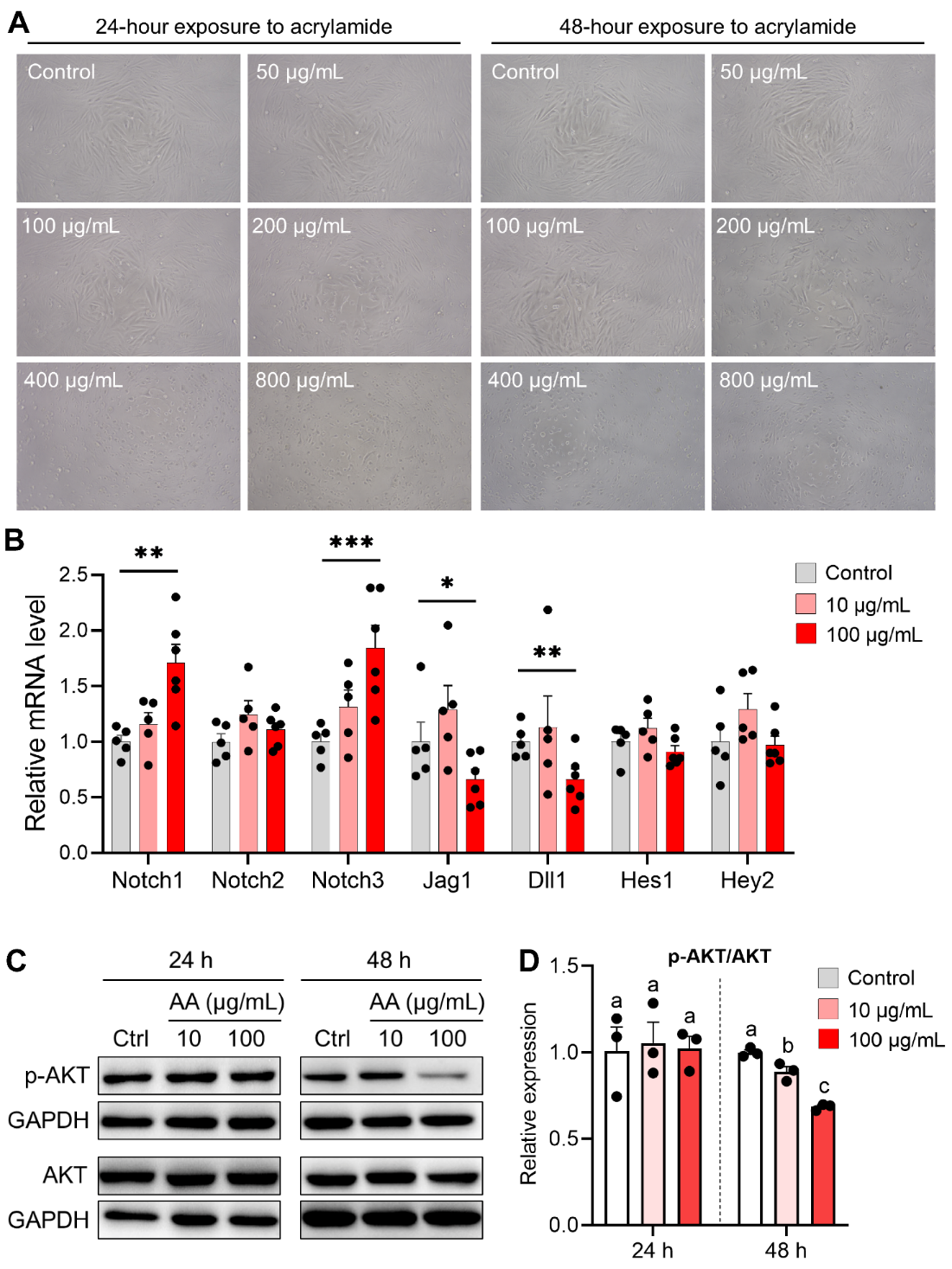
**

**Figure S10.** **Acrylamide induces H9c2 cells death and disturbs Notch signaling pathway-related genes expression in H9c2 cells.** (A) Toxic effect of acrylamide on cellular morphology in H9c2 cells after 24 h and 48 h treatment. (B) Relative mRNA levels of Notch signaling pathway-related genes in H9c2 cells with acrylamide treatment for 24 h (*n*=5‒6 per group). (C and D) Western blotting of p-AKT and AKT proteins shows that acrylamide treatment for 48 h inhibits PI3K/AKT signaling pathway in H9c2 cells (*n*=3 per group). Data are presented as the mean ± SEM. Significance was calculated using one-way ANOVA with Tukey’s post hoc test; **P* < 0.05, ***P* < 0.01, and ****P* < 0.001; groups labeled with different letters differed significantly (*P*<0.05).


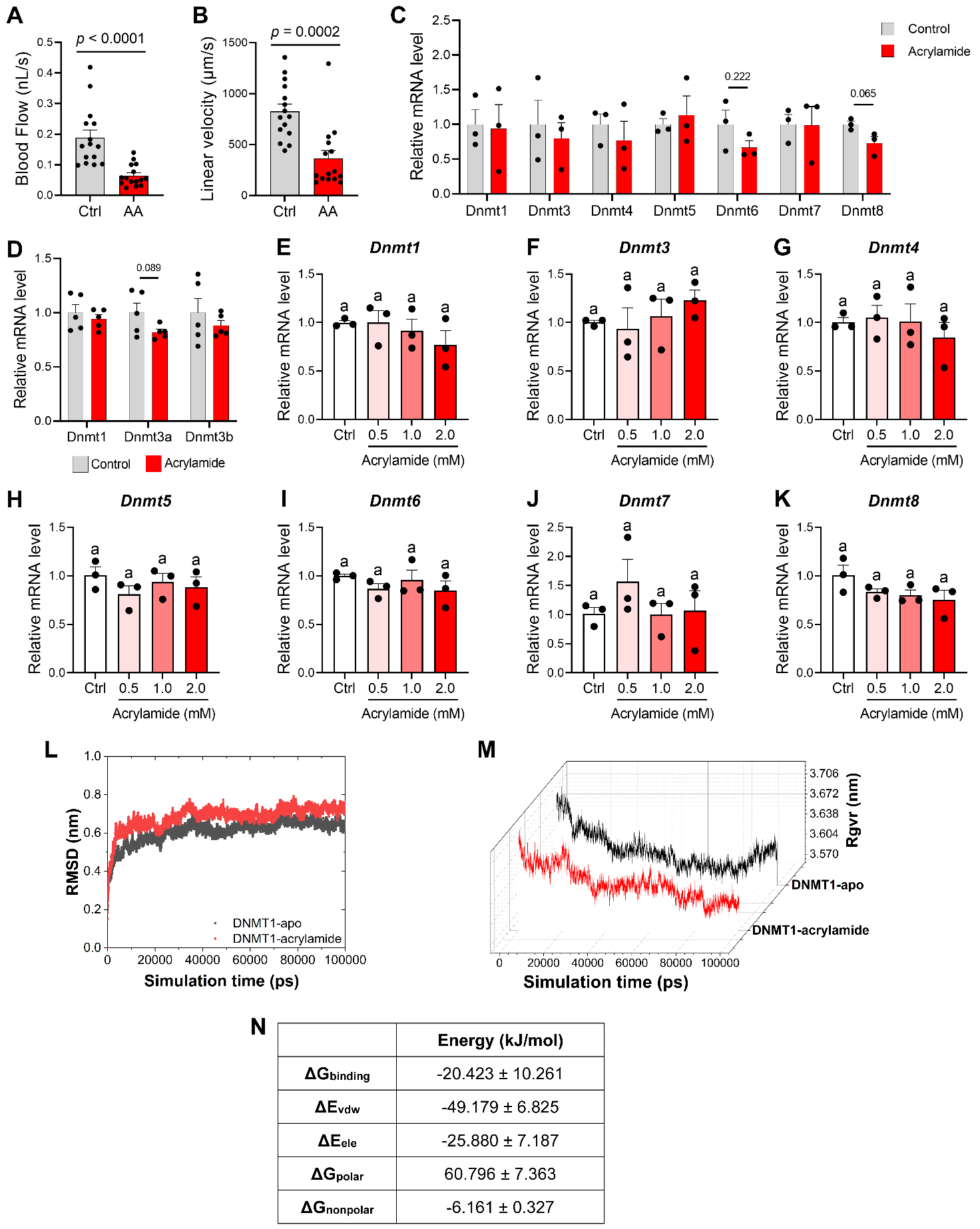


**Figure S11.** **Acrylamide exposures generates epigenetic variations via DNA methylation.** (A and B) Blood flow (nL/s) and linear velocity (μm/s) in control and acrylamide-treated (2.0 mM) zebrafish. (C) Relative mRNA levels of *Dnmt1*, *Dnmt3*, *Dnmt4*, *Dnmt5*, *Dnmt6*, *Dnmt7*, and *Dnmt8* genes in control and chronic acrylamide-treated (0.25 mM) zebrafish hearts at 180 dpf (n=3 per group). (D) Relative mRNA levels of cardiac *Dnmt1*, *Dnmt3a*, and *Dnmt3b* genes in control and chronic acrylamide-treated (0.5 mg/kg bw/day) mice (*n*=5 per group). (E‒K) Relative mRNA levels of *Dnmt1*, *Dnmt3*, *Dnmt4*, *Dnmt5*, *Dnmt6*, *Dnmt7*, and *Dnmt8* genes in control and acrylamide-treated (0.5, 1.0, and 2.0 mM) zebrafish embryos at 5 dpf (*n*=3 per group). (L) Comparison of the measured RMSD from 100 ns MD simulations. The DNMT1 apo form is colored in black, while the complex of DNMT1 with acrylamide is colored in red. (M) Comparison of the measured Rgvr from 100 ns MD simulations. The DMNT1 apo form is colored in black, the complex of DNMT1 with acrylamide is colored in red. (N) Binding free energy of acrylamide-DNMT1. Data are presented as the mean ± SEM. Significance was calculated using two-tailed *P* values by unpaired Student’s *t*-test or one-way ANOVA with Tukey’s post hoc test; groups labeled with different letters differed significantly (*P* < 0.05).

**Table S1****.** Baseline characteristics of participants across fried food consumption in the UK biobank (*n*=183,195).

|  | **Fried food consumption (serving/day)** | | |
| --- | --- | --- | --- |
| **Characteristics** | **0** | **0-1** | **≥1** |
| N | 73751 | 57265 | 52179 |
| Male (%) | 41.7 | 42.1 | 51.1 |
| race (%) |  |  |  |
| White | 94.7 | 96.3 | 94.5 |
| Non-white | 4.9 | 3.4 | 5.1 |
| Age (year) | 56.2±7.8 | 55.8±7.8 | 54.3±8.1 |
| BMI (kg/m^2^) | 26.7±4.6 | 26.6±4.5 | 27.5±4.8 |
| Physical activity (MET-h/wk) | 42.9±42.1 | 40.0±38.5 | 41.6±42.6 |
| SBP (mmHg) | 138.6±19.5 | 138.7±19.4 | 138.6±19.0 |
| DBP (mmHg) | 81.7±10.6 | 81.8±10.5 | 82.4±10.6 |
| Blood glucose (mmol/L) | 5.1±1.1 | 5.1±1.0 | 5.1±1.1 |
| Household income (£) (%) ^a^ |  |  |  |
| < 18,000 | 14.0 | 11.9 | 13.5 |
| 18,000 to 30,999 | 21.4 | 21.1 | 21.5 |
| 31,000 to 51,999 | 24.9 | 26.2 | 26.9 |
| 52,000 to 100,000 | 22.0 | 23.9 | 22.2 |
| > 100,000 | 6.6 | 7.5 | 5.9 |
| Townsend deprivation index ^b^ | -1.5±2.9 | -1.7±2.8 | -1.5±2.9 |
| Smoking (%) |  |  |  |
| Never | 58.3 | 58.7 | 55.2 |
| Previous | 33.8 | 34.6 | 34.7 |
| Current | 7.6 | 6.5 | 9.8 |
| Alcohol drinking (%) |  |  |  |
| Never or special occasions only | 17.2 | 14.1 | 16.2 |
| 1 to 3 times/month | 11.2 | 10.5 | 11.6 |
| 1 or 2 times/week | 25.1 | 25.0 | 25.1 |
| 3 or 4 times/week | 24.9 | 26.3 | 24.6 |
| daily or almost daily | 21.5 | 24.1 | 22.4 |
| History of hypertension (%) | 52.7 | 52.7 | 53.3 |
| History of high cholesterol (%) | 13.6 | 13.2 | 13.6 |
| Mediterranean Diet Score | 4.1±1.8 | 4.4±1.7 | 3.9±1.7 |
| Energy intake | 1921.8±587.3 | 2039.1±491.2 | 2301.3±671.5 |
| Vitamin use (%) | 33.7 | 32.4 | 31.1 |
| Mineral use (%) | 13.9 | 13.2 | 11.5 |

BMI=body mass index, MET=metabolic equivalent, SBP=systolic blood pressure, DBP=diastolic blood pressure.

Data are either percentage or mean ± SD unless indicated otherwise.

^a^ £1.00=$1.30, or €1.20.

^b^ The Townsend deprivation index (TDI) was an indicator of material deprivation that was calculated based on non-home ownership, non-car ownership, unemployment, and household over-crowding.

**Table S2.** Baseline characteristics of participants across fried potato consumption in the UK biobank (*n*=183,195).

|  | **Fried potato consumption (serving/day)** | | |
| --- | --- | --- | --- |
| **Characteristics** | **0** | **0-1** | **≥1** |
| N | 90937 | 59425 | 32833 |
| Male (%) | 41.9 | 43.4 | 53.6 |
| race (%) |  |  |  |
| White | 94.3 | 96.3 | 95.4 |
| Non-white | 5.3 | 3.4 | 4.3 |
| Age (year) | 56.1±7.8 | 55.6±7.8 | 53.8±8.2 |
| BMI (kg/m^2^) | 26.7±4.6 | 26.7±4.5 | 27.6±4.8 |
| Physical activity (MET-h/wk) | 42.6±41.8 | 39.8±38.7 | 42.1±43.6 |
| SBP (mmHg) | 138.5±19.5 | 138.7±19.3 | 138.6±18.8 |
| DBP (mmHg) | 81.7±10.6 | 81.9±10.5 | 82.6±10.6 |
| Blood glucose (mmol/L) | 5.1±1.1 | 5.1±1.0 | 5.1±1.2 |
| Household income (£) (%) ^a^ |  |  |  |
| < 18,000 | 13.9 | 11.7 | 14.1 |
| 18,000 to 30,999 | 21.3 | 21.1 | 21.9 |
| 31,000 to 51,999 | 25.1 | 26.5 | 27.0 |
| 52,000 to 100,000 | 22.1 | 24.0 | 21.6 |
| > 100,000 | 6.7 | 7.5 | 5.3 |
| Townsend deprivation index ^b^ | -1.5±2.9 | -1.7±2.8 | -1.5±2.9 |
| Smoking (%) |  |  |  |
| Never | 58.4 | 58.3 | 53.9 |
| Previous | 33.8 | 34.8 | 34.9 |
| Current | 7.5 | 6.7 | 10.9 |
| Alcohol drinking (%) |  |  |  |
| Never or special occasions only | 17.4 | 13.8 | 15.9 |
| 1 to 3 times/month | 11.3 | 10.4 | 11.7 |
| 1 or 2 times/week | 25.0 | 25.0 | 25.2 |
| 3 or 4 times/week | 24.7 | 26.4 | 24.5 |
| daily or almost daily | 21.5 | 24.3 | 22.5 |
| History of hypertension (%) | 52.5 | 53.0 | 53.6 |
| History of high cholesterol (%) | 13.6 | 13.4 | 13.6 |
| Mediterranean Diet Score | 4.2±1.8 | 4.4±1.7 | 3.7±1.6 |
| Energy intake | 1941.8±591.6 | 2082.0±499.5 | 2383.9±695.0 |
| Vitamin use (%) | 33.8 | 32.0 | 30.0 |
| Mineral use (%) | 14.1 | 12.7 | 10.6 |

BMI=body mass index, MET=metabolic equivalent, SBP=systolic blood pressure, DBP=diastolic blood pressure.

Data are either percentage or mean ± SD unless indicated otherwise.

^a^ £1.00=$1.30, or €1.20.

^b^ The Townsend deprivation index (TDI) was an indicator of material deprivation that was calculated based on non-home ownership, non-car ownership, unemployment, and household over-crowding.

**Table S3.** Baseline characteristics of participants across fried white meat consumption in the UK biobank (*n*=183,195).

|  | **Fried white meat consumption (serving/day)** | | |
| --- | --- | --- | --- |
| **Characteristics** | **0** | **0-1** | **≥1** |
| N | 157919 | 18120 | 7156 |
| Male (%) | 43.7 | 48.4 | 51.6 |
| race (%) |  |  |  |
| White | 95.2 | 95.5 | 92.7 |
| Non-white | 4.4 | 4.2 | 7.0 |
| Age (year) | 55.6±7.9 | 55.4±7.9 | 54.5±8.2 |
| BMI (kg/m^2^) | 26.8±4.6 | 27.0±4.6 | 27.8±4.9 |
| Physical activity (MET-h/wk) | 41.8±41.2 | 39.4±39.2 | 43.1±44.4 |
| SBP (mmHg) | 138.5±19.3 | 139.2±19.2 | 138.7±19.3 |
| DBP (mmHg) | 81.8±10.6 | 82.3±10.6 | 82.4±10.6 |
| Blood glucose (mmol/L) | 5.1±1.1 | 5.1±1.0 | 5.1±1.1 |
| Household income (£) (%) ^a^ |  |  |  |
| < 18,000 | 13.2 | 12.7 | 15.3 |
| 18,000 to 30,999 | 21.3 | 21.5 | 21.2 |
| 31,000 to 51,999 | 25.8 | 26.8 | 26.8 |
| 52,000 to 100,000 | 22.7 | 23.1 | 20.2 |
| > 100,000 | 6.8 | 6.7 | 5.0 |
| Townsend deprivation index ^b^ | -1.6±2.9 | -1.6±2.8 | -1.3±3.0 |
| Smoking (%) |  |  |  |
| Never | 57.3 | 59.4 | 57.5 |
| Previous | 34.5 | 33.3 | 32.8 |
| Current | 7.9 | 7.1 | 9.4 |
| Alcohol drinking (%) |  |  |  |
| Never or special occasions only | 15.8 | 15.8 | 19.0 |
| 1 to 3 times/month | 11.0 | 11.3 | 13.2 |
| 1 or 2 times/week | 25.0 | 25.5 | 25.7 |
| 3 or 4 times/week | 25.4 | 25.0 | 22.7 |
| daily or almost daily | 22.8 | 22.2 | 19.4 |
| History of hypertension (%) | 52.6 | 54.2 | 53.6 |
| History of high cholesterol (%) | 13.4 | 13.7 | 15.3 |
| Mediterranean Diet Score | 4.1±1.8 | 4.5±1.7 | 4.2±1.6 |
| Energy intake | 2055.0±605.0 | 2097.8±523.1 | 2241.9±769.2 |
| Vitamin use (%) | 32.6 | 31.7 | 32.5 |
| Mineral use (%) | 13.2 | 12.2 | 11.8 |

BMI=body mass index, MET=metabolic equivalent, SBP=systolic blood pressure, DBP=diastolic blood pressure.

Data are either percentage or mean ± SD unless indicated otherwise.

^a^ £1.00=$1.30, or €1.20.

^b^ The Townsend deprivation index (TDI) was an indicator of material deprivation that was calculated based on non-home ownership, non-car ownership, unemployment, and household over-crowding.

**Table S4****.** The associations between fried food, fried white meat, or fried potato consumption and heart failure risk (*n*=183,195).

|  | **Fried food consumption, servings/d** | | | | | | |
| --- | --- | --- | --- | --- | --- | --- | --- |
|  | **0** | **0-1** | | | **≥1** | | *P* for trend |
| **Fried food** |  |  | | |  | |  |
| Cases/participants | 1228/72523 | | 860/56405 | 924/51255 | |  |  |
| Model 1 ^a^ | 1.00 (ref.) | 0.91 (0.84-1.00) | | | 1.19 (1.09-1.30) | | <0.001 |
| Model 2 ^b^ | 1.00 (ref.) | 0.96 (0.88-1.05) | | | 1.10 (1.01-1.20) | | 0.046 |
| Model 3 ^c^ | 1.00 (ref.) | 0.95 (0.92-1.11) | | | 1.10 (1.01-1.20) | | 0.022 |
| **Fried white meat** |  |  | | |  | |  |
| Cases/participants | 2541/155378 | | 326/17794 | 145/7011 | |  |  |
| Model 1 ^a^ | 1.00 (ref.) | 1.10 (0.98-1.23) | | | 1.35 (1.14-1.60) | | <0.001 |
| Model 2 ^b^ | 1.00 (ref.) | 1.21 (1.02-1.24) | | | 1.21 (1.02-1.43) | | 0.007 |
| Model 3 ^c^ | 1.00 (ref.) | 1.16 (1.02-1.30) | | | 1.18 (0.99-1.40) | | 0.006 |
| **Fried potatoes** |  |  | | |  | |  |
| Cases/participants | 1540/89397 | | 854/58571 | 618/32215 | |  |  |
| Model 1 ^a^ | 1.00 (ref.) | 0.87 (0.80-0.94) | | | 1.27 (1.15-1.39) | | 0.002 |
| Model 2 ^b^ | 1.00 (ref.) | 0.90 (0.83-0.98) | | | 1.15 (1.05-1.26) | | 0.081 |
| Model 3 ^c^ | 1.00 (ref.) | 0.93 (0.85-1.01) | | | 1.15 (1.04-1.27) | | 0.047 |

^a^ Model 1 was adjusted for age and sex.

^b^ Model 2 was adjusted for age, sex, income, race center, BMI, Townsend deprivation index, smoking, alcohol use, physical activity.

^c^ Model 3 was adjusted for model 2 plus vegetable intake, fruit intake, whole grains intake, processed red meat intake, fish intake, sugar sweetened beverages intake, total energy intake (quartiles).

**Table S5.** Multivariate-adjusted HRs (95% CIs) of heart failure according to fried food consumption from sensitivity analyses.

|  | **Fried food consumption, servings/d** | | | ***P* trend** |
| --- | --- | --- | --- | --- |
|  | **0** | **0-1** | **≥1** |  |
| **Further adjustment for T2D** |  |  |  |  |
| No. of participants | 73751 | 57265 | 52179 |  |
| Heart failure cases | 1228 | 860 | 924 |  |
| Multivariable-adjusted HR (95% CI) | 1.00 (Ref.) | 0.95 (0.87-1.04) | 1.13 (1.04-1.24) | 0.014 |
| **Further adjustment for CVD** |  |  |  |  |
| No. of participants | 73751 | 57265 | 52179 |  |
| Heart failure cases | 1228 | 860 | 924 |  |
| Multivariable-adjusted HR (95% CI) | 1.00 (Ref.) | 0.99 (0.91-1.08) | 1.13 (1.04-1.24) | 0.010 |
| **Further adjustment for vitamin and mineral supplementation** |  |  |  |  |
| No. of participants | 73751 | 57265 | 52179 |  |
| Heart failure cases | 1228 | 860 | 924 |  |
| Multivariable-adjusted HR (95% CI) | 1.00 (Ref.) | 0.94 (0.86-1.03) | 1.15 (1.05-1.26) | 0.007 |
| **Further adjustment for medication use** |  |  |  |  |
| No. of participants | 73751 | 57265 | 52179 |  |
| Heart failure cases | 1228 | 860 | 924 |  |
| Multivariable-adjusted HR (95% CI) | 1.00 (Ref.) | 0.94 (0.86-1.02) | 1.11 (1.02-1.22) | 0.037 |
| **Excluding incident heart failure within 5 years** | |  |  |  |
| No. of participants | 73508 | 57095 | 52003 |  |
| Heart failure cases | 985 | 690 | 748 |  |
| Multivariable-adjusted HR (95% CI) | 1.00 (Ref.) | 0.95 (0.86-1.05) | 1.18 (1.07-1.30) | 0.004 |
| **Excluding participants with missing covariate data** |  |  |  |  |
| No. of participants | 56166 | 44797 | 40174 |  |
| Heart failure cases | 896 | 608 | 698 |  |
| Multivariable-adjusted HR (95% CI) | 1.00 (Ref.) | 0.89 (0.80-0.99) | 1.13 (1.02-1.25) | 0.049 |

**Table S6.** Characteristics of study participants by quintiles of HbAA and HbGA in NHANES 2003-2006 and 2013-2016 (*n*=10,811).

| **Characteristics** | **HbAA (pmol/g Hb)** | | | |  | **HbGA (pmol/g Hb)** | | | |
| --- | --- | --- | --- | --- | --- | --- | --- | --- | --- |
|  | **Q1** | **Q2** | **Q3** | **Q4** |  | **Q1** | **Q2** | **Q3** | **Q4** |
| **Age (years)** | 53.3±19.2 | 50.5±19.3 | 48.4±18.5 | 44.7±16.4 |  | 54.8±18.9 | 50.4±18.8 | 47.8±18.5 | 43.9±16.5 |
| **Male (%), *n* (%)** | 44.5 | 44.5 | 47.4 | 57.8 |  | 53.5 | 46.2 | 44.7 | 49.7 |
| **Education levels, *n* (%)** | |  |  |  |  |  |  |  |  |
| Under high school | 12.3 | 12.6 | 14.3 | 9.8 |  | 12.0 | 12.2 | 13.8 | 11.0 |
| High school | 22.4 | 22.3 | 21.9 | 30.1 |  | 22.5 | 20.9 | 24.1 | 29.2 |
| Above high school | 52.8 | 53.7 | 51.9 | 39.6 |  | 54.1 | 54.1 | 48.4 | 41.3 |
| **Race/ethnicity, *n* (%)** | |  |  |  |  |  |  |  |  |
| Mexican American | 13.6 | 23.0 | 26.6 | 14.1 |  | 11.2 | 20.6 | 26.5 | 19.1 |
| Non-Hispanic white | 46.2 | 48.9 | 48.2 | 53.7 |  | 46.6 | 47.2 | 47.6 | 55.7 |
| Non-Hispanic black | 20.5 | 17.3 | 16.6 | 24.8 |  | 25.0 | 19.2 | 16.5 | 18.4 |
| Others | 19.7 | 10.8 | 8.6 | 7.4 |  | 17.3 | 13.1 | 9.4 | 6.8 |
| **PIR, *n* (%)** |  |  |  |  |  |  |  |  |  |
| <1.52 | 38.6 | 34.6 | 34.0 | 45.1 |  | 36.9 | 35.2 | 37.5 | 42.9 |
| 1.52 to 3.48 | 32.9 | 31.5 | 29.4 | 31.1 |  | 33.0 | 30.7 | 29.6 | 31.6 |
| >3.48 | 28.5 | 33.9 | 36.6 | 23.8 |  | 30.2 | 34.1 | 32.9 | 25.5 |
| **Smoking Status, *n* (%)** | |  |  |  |  |  |  |  |  |
| Never | 31.9 | 30.8 | 23.9 | 5.3 |  | 29.3 | 28.2 | 23.6 | 10.8 |
| Former | 63.6 | 62.7 | 61.4 | 17.1 |  | 61.9 | 60.9 | 55.4 | 26.7 |
| Active | 4.5 | 6.6 | 14.7 | 77.6 |  | 8.8 | 10.9 | 21.1 | 62.6 |
| **Alcohol Drinking Status, *n* (%)** | | |  |  |  |  |  |  |  |
| Never | 19.3 | 16.4 | 13.9 | 6.0 |  | 16.9 | 15.3 | 14.2 | 9.1 |
| Former | 21.6 | 20.1 | 18.9 | 17.1 |  | 20.0 | 19.7 | 19.1 | 18.9 |
| Current | 53.5 | 59.4 | 62.3 | 72.4 |  | 57.7 | 60.2 | 62.1 | 67.6 |
| **Physical activity, *n* (%)** | |  |  |  |  |  |  |  |  |
| Never | 35.8 | 33.4 | 33.5 | 37.6 |  | 33.6 | 33.4 | 34.9 | 38.5 |
| Moderate | 33.0 | 32.5 | 31.7 | 28.9 |  | 31.5 | 31.7 | 31.9 | 31.1 |
| Vigorous | 27.3 | 31.5 | 32.2 | 30.6 |  | 31.1 | 32.4 | 30.5 | 27.7 |
| **BMI (kg/m^2^)** | 29.9±7.4 | 29.2±6.5 | 28.6±6.2 | 27.6±6.4 |  | 28.9±6.9 | 29.2±6.9 | 29.0±6.4 | 28.3±6.7 |
| **Diet** |  |  |  |  |  |  |  |  |  |
| Total energy intake (kcal/day) | 1939.0±764.9 | 2014.9±789.8 | 2084.3±849.5 | 2262.6±984.3 |  | 1957.2±780.6 | 2033.3±821.0 | 2077.2±838.0 | 2233.5±964.7 |
| Total fat intake (g·2000 kcal^-1^·d^-1^) | 73.8±17.1 | 74.6±16.9 | 74.5±16.7 | 74.9±17.4 |  | 73.1±17.7 | 74.6±16.5 | 74.8±16.7 | 75.3±17.2 |
| Total protein intake (g·2000 kcal^-1^·d^-1^) | 80.9±31.2 | 80.4±30.5 | 79.9±30.4 | 74.3±31.1 |  | 80.3±31.4 | 80.5±31.0 | 79.1±29.8 | 75.7±31.3 |
| Total carbohydrate intake (g·2000 kcal^-1^·d^-1^) | 249.3±49.8 | 248.8±46.9 | 248.8±46.9 | 239.9±51.2 |  | 246.7±51.2 | 247.5±46.9 | 247.2±47.2 | 245.3±50.2 |

Data are expressed as mean ± SD or numbers with percentages. HbAA, hemoglobin adducts of acrylamide; HbGA, hemoglobin adducts of glycidamide. PIR, poverty-income ratio. BMI, body mass index. HF, heart failure.

**Table S7.** Multivariate-adjusted odds ratios (95% CIs) of associations between acrylamide hemoglobin biomarkers and the prevalence of heart failure in NHANES 2003‒2006 and 2013‒2016.

| **Biomarkers** | Effect estimates (95% CI) by quantiles of metabolites | | | |  |
| --- | --- | --- | --- | --- | --- |
|  | Q1 | Q2 | Q3 | Q4 | *P*-trend |
| **HbAA (pmol/g Hb)** | <38.3 | 38.4-51.2 | 51.3-78.9 | >79.0 |  |
| Model 1 ^a^ | 1 | 0.68 (0.48-0.96) | 0.74 (0.52-1.07) | 1.19 (0.84-1.69) | 0.579 |
| Model 2 ^b^ | 1 | 0.94 (0.64-1.37) | 1.18 (0.78-1.77) | 2.01 (1.12-3.58) | 0.055 |
| Model 3 ^c^ | 1 | 0.94 (0.64-1.38) | 1.18 (0.79-1.78) | 2.05 (1.15-3.65) | 0.049 |
| **HbGA (pmol/g Hb)** | <33.6 | 33.7-47.5 | 47.6-70.4 | >70.5 |  |
| Model 1 ^a^ | 1 | 0.75 (0.53-1.06) | 0.57 (0.41-0.81) | 0.90 (0.59-1.37) | 0.195 |
| Model 2 ^b^ | 1 | 0.69 (0.47-1.01) | 0.43 (0.28-0.65) | 0.42 (0.20-0.86) | 0.001 |
| Model 3 ^c^ | 1 | 0.70 (0.48-1.01) | 0.43 (0.28-0.66) | 0.42 (0.20-0.87) | 0.001 |
| **HbAA+HbGA (pmol/g Hb)** | <73.3 | 73.3-99.6 | 99.7-149.6 | >149.7 |  |
| Model 1 ^a^ | 1 | 0.77 (0.54-1.09) | 0.64 (0.44-0.93) | 1.06 (0.75-1.49) | 0.702 |
| Model 2 ^b^ | 1 | 0.79 (0.55-1.14) | 0.64 (0.43-0.95) | 0.75 (0.44-1.26) | 0.112 |
| Model 3 ^c^ | 1 | 0.80 (0.56-1.15) | 0.65 (0.44-0.97) | 0.77 (0.46-1.30) | 0.139 |
| **HbGA/HbAA** | <0.70 | 0.70-0.87 | 0.87-1.07 | >1.07 |  |
| Model 1 ^a^ | 1 | 1.09 (0.79-1.49) | 0.63 (0.42-0.93) | 0.63 (0.44-0.91) | 0.002 |
| Model 2 ^b^ | 1 | 1.03 (0.74-1.42) | 0.56 (0.38-0.83) | 0.49 (0.34-0.73) | <0.001 |
| Model 3 ^c^ | 1 | 1.03 (0.74-1.43) | 0.56 (0.38-0.83) | 0.50 (0.34-0.74) | <0.001 |

CI, confidence interval; OR, Odd ratio; Q, quintile; PIR, poverty-income ratio; BMI, Body mass index; HbAA, haemoglobin adducts of acrylamide; HbGA, haemoglobin adducts of glycidamide.

^a^ Model 1 was adjusted for age (years), gender (male or female) and race (non-Hispanic Black, non-Hispanic White, Mexican American, or others). ^b^ Model 2 was adjusted for covariates in Model 1 plus BMI, education (under high school, high school, or above high school), PIR (< 1.52, 1.52 to 3.48, or > 3.48), physical activity (never, moderate, or vigorous), smoking (non-smoker, former smoker, or active smoker) and drinking (abstainer or active drinker) status, and log transformed HbAA (for HbGA) or HbGA (for HbAA).

^c^ Model 3 was adjusted for covariates in Model 2 plus intake of total energy.

**Table S8.** Primer pairs of selected genes in qRT-PCR analysis.

| **Models** | **Gene Name** | **Primer sequences (5′ → 3′)** |
| --- | --- | --- |
| *Danio rerio* | *β-actin* | F: AGCACGGTATTGTGACTAACTG  R: TCGAACATGATCTGTGTCATC |
| *Danio rerio* | *Nkx2.5* | F: GTCCAGGCAACTCGAACTACTC  R: AACATCCCAGCCAAACCATA |
| *Danio rerio* | *Gata4* | F: TCCAGGCGGGTGGGTTTATC  R: TGTCTGGTTCAGTCTTGATGGGTC |
| *Danio rerio* | *Tbx5a* | F: ATTCGCCGATAACAAATGG  R: CGCCTTGACGATGTGGAT |
| *Danio rerio* | *Atp2a2a* | F: ATTTACTTGTGCGGATTCTTCTAC  R: CACGATGTCTTTGGCTTTGA |
| *Danio rerio* | *P53* | F: CCCGGATGGAGATAACTTG  R: CACAGTTGTCCATTCAGCAC |
| *Danio rerio* | *Bax* | F: GGCTATTTCAACCAGGGTTCC  R: TGCGAATCACCAATGCTGT |
| *Danio rerio* | *Bcl-2* | F: TGGCGTCCCAGGTAGATAAT  R: ACCGTACATCTCCACGAAGG |
| *Danio rerio* | *Caspase-9* | F: GCCTTTCTTGATTCCCTGCG  R: TCTTGGCCTGGTTGGTCTCA |
| *Danio rerio* | *Dnmt1* | F: GAGGAGGATGTGTTGCCAGTTA  R: CCTCATTTTCCACACGCACTTT |
| *Danio rerio* | *Dnmt3* | F: TAGAGTCATGTTGAACTGGGCC  R: TCAGGTCCAGAGATTCAGGGAT |
| *Danio rerio* | *Dnmt4* | F: AAGATTTACCCTGCAGTCCCAG  R: CTCGCATACTTCTGACGCAATG |
| *Danio rerio* | *Dnmt5* | F: TTATCCACCCACTGTTCGAAGG  R: ATGACCACACAGAATGACCTCC |
| *Danio rerio* | *Dnmt6* | F: GTGTGGGGAAAGTTACGAGGAT  R: TGCTTATTGTAGGTTGGCTGGT |
| *Danio rerio* | *Dnmt7* | F: AGGCAGCTTTTCGGGATTTAGA  R: CGATTTCTTGACCATCACGAGC |
| *Danio rerio* | *Dnmt8* | F: CTTTGCCTGTTAATGAAGCCCC  R: TGTGAAGTGTCCTGTGGTTGAA |
| *Danio rerio* | *Notch 1a* | F: CGGGCCTGACGGATTCAC  R: GGACTCCAGCAGACGTTTAGC |
| *Danio rerio* | *Notch 1b* | F: AACAACCAAGATCTTTCCCATATACA  R: GCTCTAGCCATTCGCATTGAC |
| *Danio rerio* | *Dll 4* | F: CTTCACCGGACCCCTCTGT  R: TGGAAGCGGTCTTGAGTTTCTC |
| *Danio rerio* | *Jag1* | F: CCGCGTATGTTTGAAGGAGTATCAGTCG  R: CAGCACGATCCGGGTTTTGTCG |
| *Danio rerio* | *Jag2* | F: AGCCCTAGCAAAACGAGCGACG  R: GCGTGAATGTGCCGTTCGATCAA |
| *Danio rerio* | *Hey2* | F: AAGATGTGGCTCACCTACAAC  R: TGGCACCAGACGACGCAACTC |
| *Danio rerio* | *Pi3k* | F: GCAGATGGACCTTCAGATG  R: ATAACAGGGGGGATGACAG |
| *Danio rerio* | *Akt* | F: GACGGAGCCACTATGAAGAC  R: ACATCACCACCCCTAAACC |
| *Danio rerio* | *mTOR1* | F: GAAGGTGGAAGTGTTTGAGC  R: TAGCGAGCGTGTGTAGTTG |
| *Mus musculus* | *Gapdh* | F: AGGTCGGTGTGAACGGATTTG  R: TGTAGACCATGTAGTTGAGGTCA |
| *Mus musculus* | *Notch1* | F: GCTGGAAGTATTTTAGCGACGG  R: CCGCAGAAAGTGGAAGGAGT |
| *Mus musculus* | *Notch2* | F: ACAAGTGAAGTGCAGGAGAGGGG  R: CAGCGGCAGGAATAGTGAGGAG |
| *Mus musculus* | *Notch3* | F: CCGATTCTCCTGTCGTTGTCT  R: CAAGTTGTACCAGATCGGCAC |
| *Mus musculus* | *Notch4* | F: CCTATTGGCCAGCAGACAGACTA  R: GCAGTTTTTCCCCTTTTATCCCTG |
| *Mus musculus* | *Dll1* | F: CAGGGATACACACAGCAAACG  R: TTCTGTCAGGAATCTCCCCAC |
| *Mus musculus* | *Dll4* | F: CGTCGTCAGGGACAAGAATAG  R: GTTTCCTGGCGAAGTCTCTG |
| *Mus musculus* | *Jag1* | F: GCCAAACCTTGTGTAAATGCC  R: CAGTGACCCCCATTCAAGCA |
| *Mus musculus* | *Jag2* | F: AACCTGATTGGCGGCTATTAC  R: CGTACTCTAGTTCGCAATGGC |
| *Mus musculus* | *Hes1* | F: AAAGACGGCCTCTGAGCAC  R: GGTGCTTCACAGTCATTTCCA |
| *Mus musculus* | *Hey2* | F: GACTTCATGAGCATTGGATTCCG  R: CAGGTGCTGAGATGAGAGACAAG |
| *Mus musculus* | *Dnmt1* | F: CTGCTGTGGAGAAACTGGAA  R: TGATTTCCGCCTCAATGATA |
| *Mus musculus* | *Dnmt3a* | F: GAGGGAACTGAGACCCCAC  R: CTGGAAGGTGAGTCTTGGCA |
| *Mus musculus* | *Dnmt3b* | F: AGCGGGTATGAGGAGTGCAT  R: GGGAGCATCCTTCGTGTCTG |
| *Mus musculus* | *Bcl-2* | F: GGACTTCTGCAAATGCTGGACT  R: CCAGATTGGGTCCTCACACTC |
| *Mus musculus* | *Bax* | F: TTTGCTACAGGGTTTCATCCAG  R: TGTCCAGTTCATCTCCAATTCG |
| *Mus musculus* | *p53* | F: TCTTTTGTCCCTTCTCAAAA  R: CTTATTGAGGGGAGGAGAGT |
| *Mus musculus* | *Caspase3* | F: TGGAGAAATTCAAAGGACGGG  R: AGCATGGACACAATACACGGG |
| *Mus musculus* | *Caspase9* | F: AGTTCCCGGGTGCTGTCTAT  R: GCCATGGTCTTTCTGCTCAC |
| *Mus musculus* | *Akt* | F: ATGAACGACGTAGCCATTGTG  R: TTGTAGCCAATAAAGGTGCCAT |
| *Mus musculus* | *Pi3k* | F: CTGGAATGTGTGGCTGGAGT  R: AGGAGGAAGCGGTGGTCTAT |
| *Mus musculus* | *mTOR* | F: CAGTTCGCCAGTGGACTGAAG  R: GCTGGTCATAGAAGCGAGTAGAC |
| *Mus musculus* | *IL-1β* | F: CCCCAGGGCATGTTAAGGAG  R: TCTTGGCCGAGGACTAAGGA |
| *Mus musculus* | *TNF-α* | F: GACAAGGCTGCCCCGACTACG  R: CTTGGGGCAGGGGCTCTTGAC |
| *Mus musculus* | *IL-6* | F: AGTTGCCTTCTTGGGACTGA  R: CCTCCGACTTGTGAAGTGGT |
| *Mus musculus* | *Anp* | F: AGTGCGGTGTCCAACACAGAT  R: TCCTTGGCTGTTATCTTCGGTA |
| *Mus musculus* | *Bnp* | F: CCTAGCCAGTCTCCAGAGCAAT  R: CTTCCTACAACAACTTCAGTGCGT |
| *Rattus norvegicus* | *Gapdh* | F: AGTGCCAGCCTCGTCTCATA  R: GGTAACCAGGCGTCCGATAC |
| *Rattus norvegicus* | *Bmp4* | F: GAGCCATTCCGTAGTGCCAT  R: AACGACCATCAGCATTCGGT |
| *Rattus norvegicus* | *Fgf10* | F: CGGAGTTGTTGCCGTCAAAG  R: GCCACATACATTTGCCTGCC |
| *Rattus norvegicus* | *Gata4* | F: AACCAGAAAACGGAAGCCCA  R: CCCATAGTGAGATGACAGCCC |
| *Rattus norvegicus* | *Hand1* | F: ACCAGCTACATCGCCTACTTG  R: AGCCAGTGCGTCCTTTAATCC |
| *Rattus norvegicus* | *Has2* | F: AGGCGGAGGACGAGTCTATG  R: AGGCACCATACAGCCCAAAG |
| *Rattus norvegicus* | *Isl1* | F: TCCCTATGTGTTGGTTGCGG  R: TTGGCGCATTTGATCCCGTA |
| *Rattus norvegicus* | *Mef2c* | F: GCACCTACATAACATGCCGC  R: TATCTCGATGGGGTGGTGGT |
| *Rattus norvegicus* | *Nkx2.5* | F: CCCAACCGCCCCTACATTTT  R: TCTGTCTCGGCTTTGTCCAG |
| *Rattus norvegicus* | *Tbx5* | F: GATCATCACCAAGGCAGGGA  R: ATAAAGGCGACCCGGCATAG |
| *Rattus norvegicus* | *Dnmt1* | F: AAGCCAGCTATGCGACTTGGAAAC  R: ACAACCGTTGGCTTTCTGAGTGAG |
| *Rattus norvegicus* | *Dnmt3a* | F: CACCTACAACAAGCAGCCCATGTA  R: AGCCTTGCCAGTGTCACTTTCATC |
| *Rattus norvegicus* | *Dnmt3b* | F: TGTGCAGAGTCCATTGCTGTAGGA  R: GCTTCCGCCAATCACCAAGTCAAA |
| *Rattus norvegicus* | *Notch1* | F: CAATGGCACAGGGGCTATGA  R: TTAGCGGGTTGTACTGGCTG |
| *Rattus norvegicus* | *Notch2* | F: GTCTGTGATTGATCCCGCCA  R: CCATGACCAGAGCAAACCCT |
| *Rattus norvegicus* | *Notch3* | F: GCTACCTTGGCTCTGCTGAA  R: TCTCCCGGTTGGCAAAATGA |
| *Rattus norvegicus* | *Notch4* | F: GGAGGAAGAAGGGCGGTAGAG  R: TCCCTTGTCCCTGAGATAGCC |
| *Rattus norvegicus* | *Dll1* | F: TCCTTGCTTCAATGGGGGAC  R: CACAGGTAGGAGTTGCCGAG |
| *Rattus norvegicus* | *Dll4* | F: GAGGTGCGGATAACCAACGA  R: GCTGCCCACAAAGCCATAAG |
| *Rattus norvegicus* | *Jag1* | F: CTGCTTGAATGGGGGTCACT  R: CACGATTGTAGCATTGGGCG |
| *Rattus norvegicus* | *Jag2* | F: GAGTGTGCCTCTAATCCGTGT  R: GCCGCCAATCAGGTTTTTGC |
| *Rattus norvegicus* | *Hes1* | F: CAACACGACACCGGACAAAC  R: TTGGAATGCCGGGAGCTATC |
| *Rattus norvegicus* | *Hey2* | F: AGACGACCTCTGAAAGCGAC  R: TTCGATCCCGACGCCTTTTT |
